# Dysfunctional brain dynamics and their origin in Lewy body dementia

**DOI:** 10.1093/brain/awz069

**Published:** 2019-04-01

**Authors:** Julia Schumacher, Luis R Peraza, Michael Firbank, Alan J Thomas, Marcus Kaiser, Peter Gallagher, John T O’Brien, Andrew M Blamire, John-Paul Taylor

**Affiliations:** 1 Institute of Neuroscience, Newcastle University, Campus for Ageing and Vitality, Newcastle upon Tyne, UK; 2 Interdisciplinary Computing and Complex BioSystems (ICOS) research group, School of Computing, Newcastle University, Newcastle upon Tyne, UK; 3 Institute of Neuroscience, Newcastle University, The Henry Wellcome Building, Newcastle upon Tyne, UK; 4 Department of Psychiatry, University of Cambridge School of Medicine, Cambridge, UK; 5 Institute of Cellular Medicine and Newcastle Magnetic Resonance Centre, Campus for Ageing and Vitality, Newcastle upon Tyne, UK

**Keywords:** EEG microstates, functional MRI, resting state, dynamic connectivity, dementia with Lewy bodies

## Abstract

Lewy body dementia includes dementia with Lewy bodies and Parkinson’s disease dementia and is characterized by transient clinical symptoms such as fluctuating cognition, which might be driven by dysfunction of the intrinsic dynamic properties of the brain. In this context we investigated whole-brain dynamics on a subsecond timescale in 42 Lewy body dementia compared to 27 Alzheimer’s disease patients and 18 healthy controls using an EEG microstate analysis in a cross-sectional design. Microstates are transiently stable brain topographies whose temporal characteristics provide insight into the brain’s dynamic repertoire. Our additional aim was to explore what processes in the brain drive microstate dynamics. We therefore studied associations between microstate dynamics and temporal aspects of large-scale cortical-basal ganglia-thalamic interactions using dynamic functional MRI measures given the putative role of these subcortical areas in modulating widespread cortical function and their known vulnerability to Lewy body pathology. Microstate duration was increased in Lewy body dementia for all microstate classes compared to Alzheimer’s disease (*P* < 0.001) and healthy controls (*P* < 0.001), while microstate dynamics in Alzheimer’s disease were largely comparable to healthy control levels, albeit with altered microstate topographies. Correspondingly, the number of distinct microstates per second was reduced in Lewy body dementia compared to healthy controls (*P* < 0.001) and Alzheimer’s disease (*P* < 0.001). In the dementia with Lewy bodies group, mean microstate duration was related to the severity of cognitive fluctuations (ρ = 0.56, *P*_FDR_ = 0.038). Additionally, mean microstate duration was negatively correlated with dynamic functional connectivity between the basal ganglia (r = − 0.53, *P* = 0.003) and thalamic networks (r = − 0.38, *P* = 0.04) and large-scale cortical networks such as visual and motor networks in Lewy body dementia. The results indicate a slowing of microstate dynamics and disturbances to the precise timing of microstate sequences in Lewy body dementia, which might lead to a breakdown of the intricate dynamic properties of the brain, thereby causing loss of flexibility and adaptability that is crucial for healthy brain functioning. When contrasted with the largely intact microstate dynamics in Alzheimer’s disease, the alterations in dynamic properties in Lewy body dementia indicate a brain state that is less responsive to environmental demands and might give rise to the apparent slowing in thinking and intermittent confusion which typify Lewy body dementia. By using Lewy body dementia as a probe pathology we demonstrate a potential link between dynamic functional MRI fluctuations and microstate dynamics, suggesting that dynamic interactions within the cortical-basal ganglia-thalamic loop might play a role in the modulation of EEG dynamics.

## Teaser

See Jeganathan and Breakspear (doi:10.1093/brain/awz132) for a scientific commentary on this article.

Schumacher *et al.* report a marked and generalised slowing of whole-brain EEG dynamics in Lewy body dementia that distinguishes it from Alzheimer’s disease and healthy ageing. The slowing is related to a loss of dynamic interaction between basal ganglia-thalamic networks and large-scale cortical networks, and may contribute to fluctuating cognition.

## Introduction

Lewy body dementia is an umbrella term that includes both dementia with Lewy bodies and Parkinson’s disease dementia and is the second most common cause of neurodegenerative dementia in older adults after Alzheimer’s disease (McKeith, 2007). In dementia with Lewy bodies fluctuating cognition and attentional impairment are core diagnostic features (McKeith *et al.*, 2005, 2017), and these are also characteristic of Parkinson’s disease dementia (Aarsland *et al.*, 2001; Ballard *et al.*, 2002*a**,**b*; Emre *et al.*, 2007).

Fluctuating cognition in Lewy body dementia affects up to 90% of patients and appears to be qualitatively distinct from the less frequently seen fluctuations in other dementias such as Alzheimer’s disease (Bradshaw *et al.*, 2004; Lee *et al.*, 2012). In Lewy body dementia there appears to be an interruption of awareness and attention that is often associated with transient episodes of confusion and communicative difficulties. Remission to near-normal cognitive function can occur spontaneously in the absence of clear environmental triggers suggesting that fluctuating cognition in Lewy body dementia is internally driven and that dynamic changes in brain activity play a role in its aetiology (Ballard *et al.*, 2001; Sourty *et al.*, 2016). Cognitive fluctuations can occur over days and hours, but variations on shorter timescales occur, with a strong association between subsecond reaction time variability and cognitive fluctuations over longer time periods (Walker, *et al.*, 2000*a*). Often coupled with fluctuations in Lewy body dementia is marked slowing of information processing, and mental slowness, also known as bradyphrenia, a phenomenon distinct from motor slowness (Vlagsma *et al.*, 2016).

It is not clear whether there is a pathological increase or decrease in brain dynamical function associated with cognitive fluctuations. In regard to the former, early studies in Lewy body dementia posited that a second-by-second temporal instability in the spectral power of the EEG of patients with dementia with Lewy bodies was associated with the severity of cognitive fluctuations (Walker *et al.*, 2000*b*; Bonanni *et al.*, 2008). In contrast, two recent studies from our group have provided support for the counter-argument of a decrease in brain dynamical function. In the first study, we demonstrated that patients with Lewy body dementia who have marked cognitive slowing or bradyphrenia (Firbank *et al.*, 2018), had prolonged cognitive processing on functional MRI; thus the cognitive aspects of fluctuations may instead reflect a temporal mismatch between the speed of environmental change and intrinsic information processing speed. In our second study, we investigated dynamic functional MRI network function in dementia with Lewy bodies (Schumacher *et al.*, 2018*a*) and found a reduction of variability in global efficiency compared to healthy controls, which we hypothesized was due to an abnormal and temporally rigid global brain network in dementia with Lewy bodies. These findings suggest that a less dynamic brain may be apposite for the cognitive phenotype of fluctuations that occurs in Lewy body dementia. This is in alignment with the broader literature which indicates that a dynamic brain, as evidenced by temporal variability and flexibility of brain activity, is important for cognitive functioning (Deco *et al.*, 2011; Garrett *et al.*, 2013; Zalesky *et al.*, 2014) whereas less dynamic brain activity is associated with worse performance on cognitive tasks (McIntosh *et al.*, 2008; Jia *et al.*, 2014) and ageing (Guitart-Masip *et al.*, 2016; Grady and Garrett, 2018).

Brain dynamics can be assessed with different methodologies and on different timescales: while functional MRI allows the characterization of slower brain dynamics with high spatial resolution, dynamical changes on a subsecond timescale can be studied using EEG microstate analysis (Michel and Koenig, 2017). Previous research has shown that the EEG signal can be segmented into a number of short, non-overlapping, quasi-stable topographies—the microstates—that remain transiently stable for ∼80–120 ms before abruptly transitioning into a new state (Lehmann *et al.*, 1987; Khanna *et al.*, 2015; Michel and Koenig, 2017). Even though there is a large number of possible topographies in multi-channel EEG, more than 70% of its variance can be explained by only a few distinct and stereotypical topographies (Koenig *et al.*, 1999). These microstates have been described as the basic building blocks of human information processing or the ‘atoms of thought’ (Lehmann, 1990). Microstates have been shown to influence cognition and perception (Milz *et al.*, 2016; Pedroni *et al.*, 2017; Santarnecchi *et al.*, 2017) and different cognitive functions have been associated with specific microstates (Britz *et al.*, 2010). Furthermore, changes in behavioural state have been related to changes in microstate dynamics: microstates tend to get shorter in drowsiness and REM sleep compared to wakefulness (Cantero *et al.*, 1999), whereas deep sleep has been associated with an increase in overall microstate duration (Brodbeck *et al.*, 2012). The time course of occurrence of individual microstate classes does not correlate with power in specific EEG frequency bands (Britz *et al.*, 2010). However, there might be a relation between microstate dynamics and EEG oscillations with some data suggesting that increased relative power in higher frequencies might be weakly correlated with shorter overall microstate duration (Koenig *et al.*, 2002).

It has been shown that microstate temporal dynamics, especially in terms of microstate duration, are important for cognitive functioning (Van De Ville *et al.*, 2010). Furthermore, it has been suggested that investigating temporal aspects of microstate sequences can provide insight into the brain’s dynamic repertoire across different timescales. Studying microstate dynamics on a subsecond timescale can therefore provide information about brain dynamics in general with implications for fast and slow dynamic processes (Van De Ville *et al.*, 2010).

Thus, interrogation of microstate dynamics in Lewy body dementia may provide a novel perspective in understanding the basis of cognitive fluctuations and more broadly the Lewy body dementia cognitive phenotype. These investigations form the first part of the paper.

In the second part, we address potential mechanisms that might be related to microstate transition and their disruption in Lewy body dementia. While there is evidence for a relation between specific microstates and the well known resting state networks that can be obtained from functional MRI (Britz *et al.*, 2010; Musso *et al.*, 2010; Custo *et al.*, 2017), it remains unclear which processes in the brain are related to the abrupt global transitions between different microstates (Michel and Koenig, 2017). However, subcortical-cortical networks represent one putative system which could globally alter brain dynamics given their significant and widespread cortico-petal connectivity. In particular, both the thalamus and the basal ganglia have extensive connections to various parts of the cortex and form part of the cortical-basal ganglia-thalamic loop, which is an important contributor to large-scale network communication within the brain (Bell and Shine, 2016). The thalamus has been suggested to play a role in modulating the cortical EEG signal (Lopes da Silva, 1991) and its activity has been shown to relate to cortical microstate characteristics (Schwab *et al.*, 2015). From a Lewy body dementia perspective, structural and functional abnormalities of the thalamus are a common feature in Lewy body diseases (Watson *et al.*, 2017). In particular, microstructural changes and cholinergic imbalance in the thalamus have been suggested to play a role in the aetiology of cognitive fluctuations in dementia with Lewy bodies (Pimlott *et al.*, 2006; Delli Pizzi, *et al.*, 2015*a*). Similarly, dopaminergic dysfunction of the basal ganglia is a hallmark of Lewy body diseases (McKeith *et al.*, 2007) and aberrant functional connectivity of the basal ganglia network has been found in dementia with Lewy bodies and Parkinson’s disease (Szewczyk-Krolikowski *et al.*, 2014; Rolinski *et al.*, 2015; Schumacher *et al.*, 2018*b*). Both the basal ganglia and the thalamic networks are therefore potential candidate networks whose dynamic interaction with cortical networks might influence microstate dynamics in Lewy body dementia.

In summary, we sought to test two main hypotheses: (i) a less dynamic brain, as evidenced by slowing of microstate dynamics is a feature of Lewy body dementia, which is related to the cognitive phenotype, and in particular, cognitive fluctuations; and (ii) disturbances in microstate dynamics in Lewy body dementia would be contingent upon a loss of dynamics within cortical-basal ganglia-thalamic connections.

## Materials and methods

### Participants

The study involved 96 participants who were over 60 years of age. Forty-six were diagnosed with probable Lewy body dementia (25 dementia with Lewy bodies and 21 Parkinson’s disease dementia), 32 with probable Alzheimer’s disease, and 18 were age-matched healthy controls with no history of psychiatric or neurological illness. Patients were recruited from the local community-dwelling population who had been referred to old age psychiatry and neurology services between 2010 and 2014. The study was approved by the local ethics committee and written informed consent was obtained from all participants. Dementia diagnoses were performed independently by two experienced clinicians in alignment with the consensus criteria for probable dementia with Lewy bodies (McKeith *et al.*, 2005), Parkinson’s disease dementia (Emre *et al.*, 2007), and Alzheimer’s disease (McKhann *et al.*, 2011). Patients who were taking dopaminergic medication were assessed in the ‘ON’ motor state.

All participants underwent a detailed neurological and neuropsychiatric assessment. Tests that were relevant to the present study included the Mini-Mental State Examination (MMSE) and Cambridge Cognitive Examination (CAMCOG) as measures of global cognition, the Unified Parkinson’s Disease Rating Scale (UPDRS) part III for the assessment of Parkinsonian motor problems, and the Neuropsychiatric Inventory (NPI) hallucination subscale, which was specifically focussed on visual hallucination occurrence. For the assessment of cognitive fluctuations we used the Clinician Assessment of Fluctuation (CAF, Walker *et al.*, 2000*c*), which provides a global measure of the duration and frequency of cognitive fluctuations and the Mayo Fluctuation Scale (Ferman *et al.*, 2004), which has two major phenotypic dimensions of fluctuations: a cognitive-attention subscale and an arousal-alertness subscale (Bliwise *et al.*, 2014).

### EEG acquisition and preprocessing

Resting state EEG recordings were acquired from all participants using Waveguard caps (ANT Neuro) comprising 128 sintered Ag/AgCl electrodes that were placed according to the 10–5 system. Participants were seated during the recording and were instructed to remain awake, but keep their eyes closed. Electrode impedance was kept below 5 kΩ and 150 s of continuous EEG data were recorded at a sampling frequency of 1024 Hz. The ground electrode was attached to the right clavicle and all EEG channels were referenced to Fz during recording.

Preprocessing of EEG data was performed blinded to group membership and methods applied were the same as described in Peraza *et al.* (2018). Briefly, data were filtered between 0.3 to 54 Hz using a second order Butterworth filter, noisy EEG segments with artefacts affecting all channels were deleted, and independent component analysis was used for artefact removal. Data were then recomputed against the average reference, bandpass filtered between 2 and 20 Hz, and split into non-overlapping epochs of 2 s. For each participant the first 30 2-s long artefact-free epochs were selected for the microstate analysis. Four Alzheimer’s disease and five Parkinson’s disease dementia patients with <30 artefact-free epochs were excluded from further analysis. This resulted in 18 healthy controls, 27 patients with Alzheimer’s disease, and 42 patients with Lewy body dementia (25 dementia with Lewy bodies and 17 Parkinson’s disease dementia) for further analysis.

### Microstate analysis

**Figure 1:**
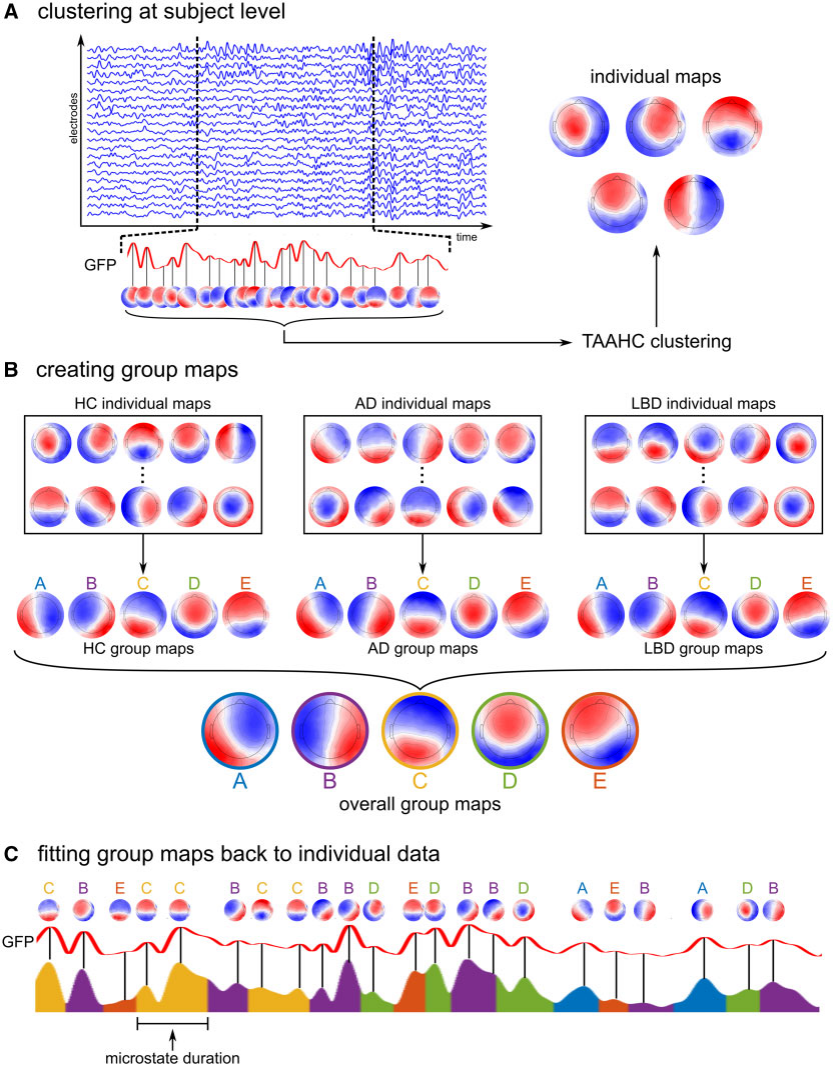
**Microstate analysis methods.** (**A**) For each subject, data at global maxima of the GFP are clustered using the TAAHC algorithm to obtain individual microstate maps. (**B**) The individual maps are combined to obtain group maps within each clinical group using a permutation algorithm. (**C**) Group maps are fit back to the data at GFP peaks assigning each GFP peak to the microstate class with the highest topographical correlation. Microstates in-between GFP peaks are interpolated. AD = Alzheimer’s disease; HC = healthy controls; LBD = Lewy body dementia; TAAHC = topographic atomize and agglomerate hierarchical clustering.

The microstate analysis was conducted using the Cartool software (Brunet *et al.*, 2011) and functions from the EEGLAB plugin for microstates (http://www.thomaskoenig.ch/index.php/software/microstates-in-eeglab) in MATLAB R2017a. As a first step, the global field power (GFP) was calculated, which is equivalent to the spatial standard deviation of the average-referenced signal across all electrodes and whose local maxima represent instants of highest field strength (Lehmann and Skrandies, 1980). EEG topographies tend to remain stable during periods of high GFP and change rapidly around the local minima of the GFP (Lehmann *et al.*, 1987). Thus, topographies at GFP peaks are representative of topographies at surrounding time points and restricting the microstate analysis to these GFP peaks provides optimal topographic signal-to-noise ratios (Lehmann *et al.*, 1987). For each subject separately, topographies at GFP peaks were subjected to a topographic atomize and agglomerate hierarchical clustering (TAAHC) algorithm (Murray *et al.*, 2008) (Fig. 1A). The optimal number of microstate classes *k* was determined for each participant individually using the meta-criterion described in Custo *et al.* (2017), testing the entire range of 1 to 12 classes. The individual maps were then averaged across all participants within each group using a permutation algorithm (Koenig *et al.*, 1999) and overall mean maps across all participants were obtained by averaging the group-specific average maps across groups (Fig. 1B).

The group microstate maps were then fit back to the original data at GFP peaks assigning each GFP peak to one microstate class based on the maximal spatial correlation between topographies (Fig. 1C). Microstate labels for data points between GFP peaks were interpolated with microstates starting and ending halfway between two GFP peaks. Potentially truncated microstates at the beginning and end of each epoch were excluded from the analysis. Microstate duration was then calculated as the time during which all successive maps were assigned to the same microstate. Additionally, the mean number of occurrences of each microstate class per second (microstate occurrence) and the percentage of total analysis time covered by each microstate (microstate coverage) were calculated.

Furthermore, transition probabilities were analysed. Details are available in the Supplementary material.

### Microstate statistics

The topographies of the different microstate classes were compared between the groups using TANOVA (topographical analysis of variance) implemented in the Ragu software (Koenig *et al.*, 2011). For this, a non-parametric randomization test was performed on global map dissimilarity with a within-subject factor of microstate class and a between-subject factor of group.

Microstate duration, occurrence, coverage, and observed transition probabilities were compared between the groups using separate MANOVAs (multivariate analyses of variance) in SPSS (Statistical Package for the Social Sciences v23, IBM). In the case of an overall significant test, follow-up univariate ANOVAs were performed to determine which microstate classes showed group differences followed by *post hoc* tests with Bonferroni-correction for multiple comparisons.

Spearman’s correlations between mean microstate duration and the Mayo fluctuation scale (overall score, cognitive subscore, and arousal subscore) were tested in the combined Lewy body dementia group as well as in the two subgroups separately. *P*-values were FDR (false discovery rate)-corrected for multiple comparisons. To check whether these correlations were influenced by dopaminergic medication, a linear regression analysis was also performed adding levodopa equivalent daily dose (LEDD, Tomlinson *et al.*, 2010) as a covariate in the model. Supplementary correlation analyses were performed for the CAF score in the Lewy body dementia group and for global cognitive scores (MMSE and CAMCOG) across the dementia groups.

As an additional exploratory analysis, Spearman’s correlations were calculated in the Lewy body dementia group between Mayo fluctuation scores and microstate duration for each microstate class separately.

### Frequency analysis

A consistent finding from previous EEG studies in Lewy body dementia is the observation of a general slowing of oscillatory EEG activity as evidenced by a slowing of the dominant frequency in posterior regions compared to healthy controls and Alzheimer’s disease patients (Cromarty *et al.*, 2015; Bonanni *et al.*, 2016; Peraza *et al.*, 2018; Stylianou *et al.*, 2018). To investigate the relationship between this general EEG slowing and microstate temporal characteristics, we calculated the Pearson’s correlation between dominant frequency and mean microstate duration and the number of GFP peaks per second in the Lewy body dementia group. To assess posterior EEG slowing, dominant frequency was estimated by averaging the signal from all occipital channels (PO9, PO7, POO9h, PO5, O1, PO3, POO3h, OI1h, POz, Oz, PO4, POO4h, PO6, O2, OI2h, PO8, POO10h, PO10) and estimating power spectral density with Welch’s periodogram (Peraza *et al.*, 2018). Dominant frequency was defined as the frequency bin in the power spectrum with the highest power between 4 and 15 Hz, and for each participant the mean dominant frequency across the whole EEG timecourse was estimated. In addition to only using occipital channels, the dominant frequency analysis was repeated using all channels.

Additionally, the power from all electrodes was estimated for different frequency bands (delta: 0.5–4 Hz, theta: 4–5.5 Hz, high-theta: 5.5–8 Hz, alpha: 8–13, and beta: 13–30) and correlated with mean microstate duration and number of GFP peaks per second.

### Functional MRI dynamic connectivity

Resting state functional MRI was recorded from a subset of the participants included in the EEG study (non-concurrent, performed between 1 and 3 weeks apart). This subset comprised 12 healthy controls, 14 patients with Alzheimer’s disease, and 29 patients with Lewy body dementia (17 dementia with Lewy bodies and 12 Parkinson’s disease dementia). Details on acquisition and preprocessing can be found in Schumacher *et al.* (2018*a*). The dynamic connectivity analysis followed the same steps as described by Allen *et al.* (2014) and Schumacher *et al.* (2018a). Briefly, dynamic connectivity was calculated using a sliding-window approach to assess how the functional connectivity between different resting state networks changed over time (see Supplementary material for a more detailed description). In the present analysis, given our *a priori* hypothesis, we focussed on dynamic connectivity between two subcortical networks (basal ganglia and thalamic networks) and all other networks (see Supplementary Table 12 for a list of all included networks and Fig. 5 for a depiction of the network maps).

### Combining EEG microstates and dynamic functional MRI connectivity

The mean variability of connectivity (standard deviation over time) between the two subcortical networks of interest—basal ganglia and thalamus—and all other networks was calculated and correlated with mean microstate duration in each group separately using Pearson’s correlations. To assess which of the individual network connections contributed most to the overall correlation, we correlated mean microstate duration with the dynamic connectivity of each connection separately, correcting the resulting *P*-values for multiple comparisons using FDR correction. The same analysis was performed for the duration of each microstate class separately (Supplementary Tables 17 and 18).

### Data availability

The data that support the findings of this study are available from the corresponding author, upon reasonable request.

## Results

### Demographics

Supplementary Table 1 shows a comparison of clinical symptoms between the two Lewy body groups. Both subgroups were matched in terms of age, gender, overall cognition, the percentage of patients taking cholinesterase inhibitors, and cognitive fluctuation and visual hallucination severity. More Parkinson’s disease dementia patients were taking dopaminergic medication and they had worse Parkinsonism than the patients with dementia with Lewy bodies. Dementia with Lewy bodies and Parkinson’s disease dementia patients were combined into one Lewy body dementia group as preliminary analyses showed that there were no group differences with respect to microstate characteristics (Supplementary Table 2).

Healthy control, Alzheimer’s disease, and Lewy body dementia participants were similar in age and gender (Table 1). Additionally, the two dementia groups did not differ significantly in terms of dementia duration. However, the Lewy body dementia group was significantly less impaired in terms of overall cognition (MMSE and CAMCOG) compared to the Alzheimer’s disease group. The percentage of patients taking cholinesterase inhibitors did not differ between the dementia groups whereas, as expected, significantly more Lewy body dementia patients were taking dopaminergic medication compared to the Alzheimer’s disease group. The Lewy body dementia patients were more impaired than the Alzheimer’s disease patients with respect to the core Lewy body dementia symptoms of parkinsonism, cognitive fluctuations, and visual hallucinations.

**Table 1:** Demographic and clinical variables

	Healthy controls (*n* = 18)	Alzheimer’s disease (*n* = 27)	LBD (*n* = 42)	Between-group differences
Male: female	11:7	20:7	36:6	χ^2 ^= 4.5, *P* = 0.11^a^
Age	76.3 (5.5)	74.9 (7.0)	74.8 (6.4)	*F*(2,84) = 0.35, *P* = 0.70^b^
AChEI	-	25	36	χ^2 ^= 0.76, *P* = 0.38^c^
PD meds	-	1	29	χ^2 ^= 28.6, *P* < 0.001^c^
Duration	-	3.9 (2.1)^f^	3.2 (2.1)^g^	U = 399, *P* = 0.12^d^
MMSE	29.2 (0.9)	20.7 (4.3)	23.1 (3.7)	*t*(67) = 2.51, *P* = 0.01^e^
CAMCOG	96.7 (3.7)	67.4 (15.7)	75.7 (11.1)	*t*(67) = 2.57, *P* = 0.01^e^
UPDRS III	1.3 (1.5)	2.4 (3.0)	20.4 (8.5)	*t*(67) = 10.6, *P* < 0.001^e^
CAF total	-	0.38 (0.98)^g^	5.0 (4.3)^h^	*t*(64) = 5.31, *P* < 0.001^e^
Mayo total	-	9.4 (4.7)^g^	14.0 (5.7)^h^	*t*(64) = 3.41, *P* = 0.001^e^
Mayo cogn	-	1.9 (1.8)^g^	2.8 (1.8)^h^	*t*(64) = 2.06, *P* = 0.043^e^
NPI total	-	6.8 (6.6)^g^	14.3 (10.5)^i^	*t*(65) = 3.23, *P* = 0.002^e^
NPI hall	-	0.04 (0.20)^g^	1.9 (2.0)^i^	*t*(65) = 4.90, *P* < 0.001^e^

Values are mean (SD).

AChEI = number of patients taking acetylcholinesterase inhibitors; CAF total = Clinician Assessment of Fluctuation total score; CAMCOG = Cambridge Cognitive Examination; Duration = duration of cognitive symptoms in years; LBD = Lewy body dementia; Mayo total = Mayo Fluctuations Scale; Mayo cognitive = Mayo Fluctuation cognitive subscale; MMSE = Mini-Mental State Examination; PD meds = number of patients taking dopaminergic medication for the management of Parkinson’s disease symptoms; UPDRS III = Unified Parkinson’s Disease Rating Scale III (motor subsection); NPI = Neuropsychiatric Inventory; NPI hall = NPI hallucination subscore.

^a^Chi-square test healthy controls, Alzheimer’s disease, Lewy body dementia.

^b^One-way ANOVA healthy controls, Alzheimer’s disease, Lewy body dementia.

^c^Chi-square test Alzheimer’s disease, Lewy body dementia.

^d^Mann Whitney U-test Alzheimer’s disease, Lewy body dementia.

^e^Student’s *t*-test Alzheimer’s disease, Lewy body dementia.

^f^ *n* = 25, ^g^*n* = 26, ^h^*n* = 40, ^i^*n* = 41.

To ensure that the difference in overall cognition between the two dementia groups did not influence the results, all analyses described below were rerun with Alzheimer’s disease and Lewy body dementia subgroups that were matched for MMSE and CAMCOG (Supplementary material).

Demographics for those participants that were included in the combined EEG-functional MRI analysis are shown in Supplementary Table 13. All three groups were matched for age and gender, while the two dementia groups were matched in terms of overall cognition.

### Cluster evaluation

The optimal number of microstate classes for each participant was determined to be between four and eight. The median within each clinical group as well as the overall median was five, with no significant differences between the groups [Kruskal-Wallis ANOVA, *H*(2) = 0.93, *P* = 0.63]. The number of microstate classes was therefore set to five for all subsequent analyses.

Across all participants the mean global explained variance of five microstate classes was 70% [standard deviation (SD) = 6%]. The mean and standard deviation in each group was 71% (SD = 8%) for healthy controls, 68% (SD = 5%) for Alzheimer’s disease, and 71% (SD = 5%) for Lewy body dementia. A univariate ANOVA showed that there were no significant group differences [*F*(2,84) = 3.01, *P* = 0.06]. Nevertheless, as there was a trend for lower explained variance in the Alzheimer’s disease group compared to Lewy body dementia, group comparisons of microstate characteristics were repeated including global explained variance as covariate (Supplementary material). This analysis indicated that including this covariate did not change the overall significance of the results regarding temporal microstate characteristics.

### Microstate topographies

**Figure 2:**
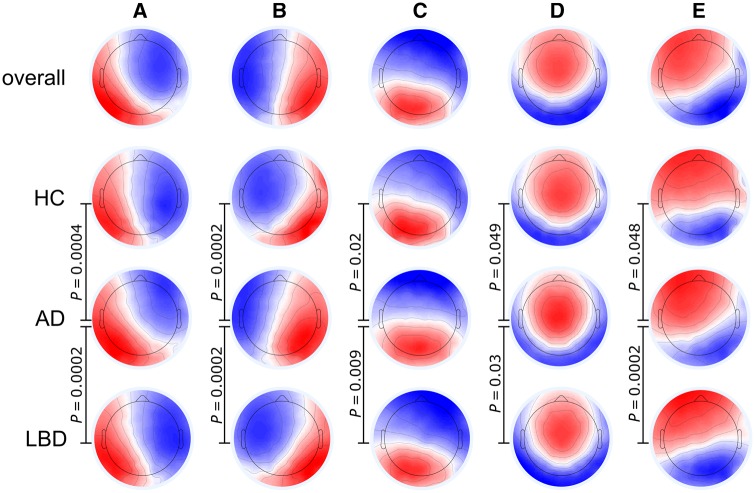
**Microstate class topographies.** *P*-values result from comparing the group topographies between groups using TANOVA. For the comparison between healthy controls and Lewy body dementia all *P* -values were >0.1. AD = Alzheimer’s disease; HC = healthy controls; LBD = Lewy body dementia.

Group microstate maps and the overall maps across all participants are shown in Fig. 2. Microstate classes A to D corresponded well to the canonical microstate maps that have been reported in the literature (Michel and Koenig, 2017). There was an additional microstate class E that resembles a slightly lateralized version of class D and might be comparable to the deviant microstate topography of class C that has been described by Grieder *et al.* (2016) in a group of patients with semantic dementia.

The overall TANOVA revealed a significant main effect of microstate class (*P* = 0.0002) and a main effect of group (*P* = 0.0002), but no interaction between the two factors (*P* = 0.45). Follow-up TANOVAs for each microstate class showed that the Alzheimer’s disease topographies were different from both the healthy controls and Lewy body dementia topographies for all microstate classes (Fig. 2 and Supplementary Tables 6 and 7). In contrast, there were no significant differences between healthy controls and Lewy body dementia topographies for any microstate class.

### Microstate temporal characteristics

The mean number of GFP peaks per second was 21.3 in healthy controls, 19.7 in Alzheimer’s disease, and 17.2 in Lewy body dementia. There was a significant difference between groups [univariate ANOVA, *F*(2,84) = 26.6, *P* < 0.001] with Bonferroni-corrected *post hoc* tests demonstrating that the number of GFP peaks was lower in Alzheimer’s disease compared to controls (*P* = 0.034), and lower in Lewy body dementia compared to controls (*P* < 0.001) and Alzheimer’s disease (*P* < 0.001). To confirm that the group differences in microstate duration and occurrence reported below were not merely due to differences in the number of GFP peaks we repeated the microstate analysis, this time fitting the group maps to all individual data points instead of restricting the fitting procedure to data at GFP peaks (Supplementary material).

**Figure 3:**
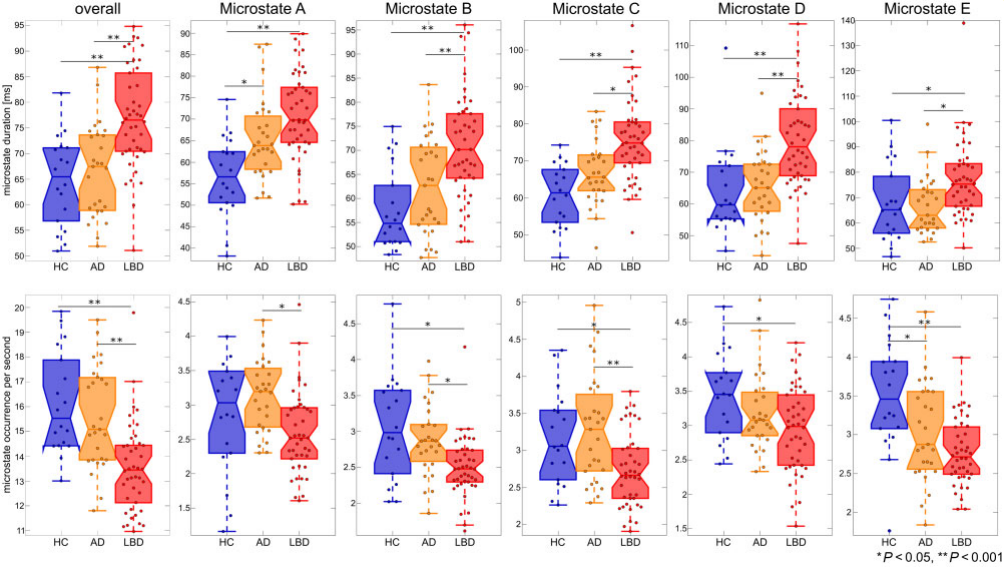
**Temporal microstate characteristics.** Group comparison of microstate duration and occurrence per second overall and for each microstate class separately. *P*-values result from pairwise *post hoc* tests following univariate ANOVAs. See Tables 2 and 3 for detailed information on statistics. AD = Alzheimer’s disease; HC = healthy controls; LBD = Lewy body dementia.

Across all microstate classes, mean microstate duration was 65 ms in controls, 67 ms in Alzheimer’s disease patients, and 77 ms in the Lewy body dementia group. A univariate ANOVA followed by *post hoc* group comparisons showed that mean microstate duration was increased in Lewy body dementia compared to controls and Alzheimer’s disease with no significant difference between Alzheimer’s disease and controls (Fig. 3 and Table 2). Correspondingly, the mean number of unique microstate occurrences per second was 16 in controls, 15.5 in Alzheimer’s disease, and 13.5 in Lewy body dementia. Univariate ANOVA and *post hoc* tests showed that the number of unique microstate occurrences per second was significantly decreased in Lewy body dementia compared to controls and Alzheimer’s disease with no significant difference between Alzheimer’s disease and controls (Fig. 3 and Table 3). These results did not change when fitting all individual time points (Supplementary material).

**Table 2:** Microstate duration for microstate classes A to E and the three clinical groups, and results from group comparison using univariate ANOVAs and pairwise *post hoc* tests

	Healthy controls	Alzheimer’s disease	Lewy body dementia	ANOVA	*Post hoc* (*P*-value)		
					HC-AD	HC-LBD	AD-LBD
Mean	64.7	66.6	77.0	*F*(2,84) = 15.5,	1.0	<0.001	<0.001
	[60.2,69.1]	[63.0,70.3]	[74.2,79.9]	*P* < 0.001			
A	56.6	65.4	71.0	*F*(2,84) = 14.2	0.01	<0.001	0.06
	[52.1,61.1]	[61.7,69.1]	[68.0,73.9]	*P* < 0.001			
B	57.6	62.3	71.0	*F*(2,84) = 12.9	0.38	<0.001	0.003
	[52.9,62.4]	[58.5,66.2]	[67.9,74.1]	*P* < 0.001			
C	60.8	66.9	75.7	*F*(2,84) = 16.0	0.14	<0.001	0.002
	[56.1,65.4]	[63.1,70.7]	[72.7,78.8]	*P* < 0.001			
D	64.2	65.6	80.1	*F*(2,84) = 13.9	1.0	<0.001	<0.001
	[57.9,70.4]	[60.5,70.7]	[76.0,84.2]	*P* < 0.001			
E	67.6	66.7	77.2	*F*(2,84) = 5.7	1.0	0.05	0.01
	[61.0,74.2]	[61.3,72.1]	[72.9,81.5]	*P* = 0.005			

Values are mean [95% confidence interval, CI].

AD = Alzheimer’s disease; HC = healthy controls; LBD = Lewy body dementia.

*Post hoc P*-values are Bonferroni-corrected for multiple comparisons.

**Table 3:** Microstate occurrence per second for microstate classes A to E and the three clinical groups, and results from group comparison using univariate ANOVAs and pairwise *post hoc* tests

	Healthy controls	Alzheimer’s disease	Lewy body dementia	ANOVA	*Post hoc* (*P*-value)		
					HC-AD	HC-LBD	AD-LBD
Mean	16.1	15.5	13.5	*F*(2,84) = 15.1	0.99	<0.001	<0.001
	[15.2,17.0]	[14.8,16.3]	[12.9,14.1]	*P* < 0.001			
A	3.0	3.1	2.6	*F*(2,84) = 5.6	1.0	0.17	0.005
	[2.6,3.3]	[2.9,3.4]	[2.4,2.8]	*P* = 0.005			
B	3.1	2.9	2.5	*F*(2,84) = 8.3	0.87	0.001	0.01
	[2.8,3.3]	[2.7,3.1]	[2.3,2.7]	*P* < 0.001			
C	3.2	3.3	2.7	*F*(2,84) = 8.2	1.0	0.03	<0.001
	[2.9,3.4]	[3.1,3.5]	[2.5,2.9]	*P* < 0.001			
D	3.4	3.2	3.0	*F*(2,84) = 4.3	0.58	0.02	0.31
	[3.1,3.7]	[3.0,3.4]	[2.8,3.1]	*P* = 0.016			
E	3.5	3.0	2.8	*F*(2,84) = 10.8	0.02	<0.001	0.16
	[3.2,3.8]	[2.8,3.3]	[2.6,2.9]	*P* < 0.001			

Values are mean [95% confidence interval, CI]. *Post hoc P*-values are Bonferroni-corrected for multiple comparisons.

AD = Alzheimer’s disease; HC = healthy controls; LBD = Lewy body dementia.

Multivariate ANOVAs followed by *post hoc* univariate ANOVAs were conducted to test for group differences in mean microstate duration and occurrence for microstate classes A to E (Fig. 3 and Tables 2 and 3). Microstate A duration was increased in both dementia groups compared to controls with a trend for a further increase in Lewy body dementia compared to Alzheimer’s disease. Occurrence of microstate A was reduced in Lewy body dementia compared to controls and Alzheimer’s disease with no difference between controls and Alzheimer’s disease. The other microstates (B to E) showed similar patterns in terms of duration with increased duration in Lewy body dementia compared to Alzheimer’s disease and controls and no difference between Alzheimer’s disease and controls. The occurrence of microstates B and C was decreased in Lewy body dementia compared to controls and Alzheimer’s disease with no difference between controls and Alzheimer’s disease. In contrast, microstate D occurrence was only reduced in Lewy body dementia compared to controls, but there was no difference between controls and Alzheimer’s disease and between the dementia groups. The occurrence of microstate E was reduced in both dementia groups compared to controls with no difference between the dementia groups.

Repeating the group comparison analyses with matched dementia groups did not change the overall results, but enhanced some of the differences between the Alzheimer’s disease and Lewy body dementia groups (Supplementary Tables 4 and 5).

Microstate coverage, i.e. the percentage of total analysis time spent within each microstate, was not different between the groups [MANOVA, *F*(8,164) = 1.79, *P* = 0.08]; this was further confirmed with univariate *post hoc* analysis (Supplementary Table 8).

Transition probabilities were found to be non-random in all three groups and there were no group differences in transition probabilities (Supplementary material).

### Clinical correlations

**Figure 4:**
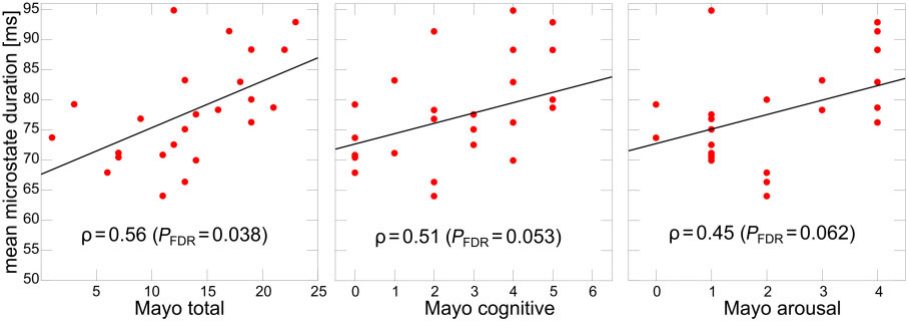
**Clinical correlations.** Spearman’s correlations between mean microstate duration and Mayo fluctuation scores in the dementia with Lewy bodies group. *P*-values are FDR-corrected for multiple comparisons. Mayo arousal = Mayo Fluctuations arousal subscale; Mayo total = Mayo Fluctuations Scale; Mayo cognitive = Mayo Fluctuation cognitive subscale.

Figure 4 shows results from Spearman’s correlations between the Mayo fluctuation scales and mean microstate duration in the dementia with Lewy bodies patients with *P*-values FDR-corrected for multiple comparisons. There was a positive correlation between mean microstate duration and the Mayo total score in the combined Lewy body dementia group (ρ = 0.36, *P*_FDR_ = 0.06), which was mainly driven by the dementia with Lewy bodies patients (ρ = 0.56, *P*_FDR_ = 0.038) and was not present in the Parkinson’s disease dementia group (*P* > 0.1). A similar pattern was observed for the Mayo cognitive subscale whereas correlations were weaker for the Mayo arousal subscale (Fig. 4 and Supplementary Table 10). There were non-significant trend associations with CAF total score and CAF duration score with mean microstate duration in the dementia with Lewy bodies group (*P* ≤ 0.10). A linear regression analysis with LEDD as covariate indicated that the covariate did not have a significant effect on the correlation between the Mayo total score and mean microstate duration in the dementia with Lewy bodies group (*P* = 0.55).

When considering each microstate class separately in the Lewy body dementia group, the Mayo total score and the Mayo cognitive subscore were positively correlated with duration of microstates A–C and E while there was no significant correlation with duration of microstate D (Supplementary Table 11). Again, these correlations were mainly driven by the dementia with Lewy bodies group and were not significant in the Parkinson’s disease dementia patients.

### Frequency analysis

There were weak, but non-significant negative correlations between dominant frequency and mean microstate duration in the Lewy body dementia group when estimating dominant frequency only from occipital channels (r = −0.25, *P* = 0.11) or using all electrodes (r = −0.25, *P* = 0.12). In contrast, the number of GFP peaks per second was significantly positively correlated with dominant frequency from occipital electrodes (r = 0.41, *P* = 0.007) and when considering all electrodes (r = 0.36, *P* = 0.02).

Power within the delta band was positively correlated with mean microstate duration (r = 0.35, *P* = 0.02) and negatively correlated with the number of GFP peaks per second (r = −0.6, *P* < 0.001) (Supplementary Table 12). In contrast, beta band power was negatively correlated with mean microstate duration (r = −0.58, *P* < 0.001) and positively correlated with the number of GFP peaks per second (r = 0.71, *P* < 0.001). Additionally, alpha power was positively correlated with the number of GFP peaks per second (r = 0.45, *P* = 0.003).

### Relation between dynamic connectivity and microstate duration

**Figure 5:**
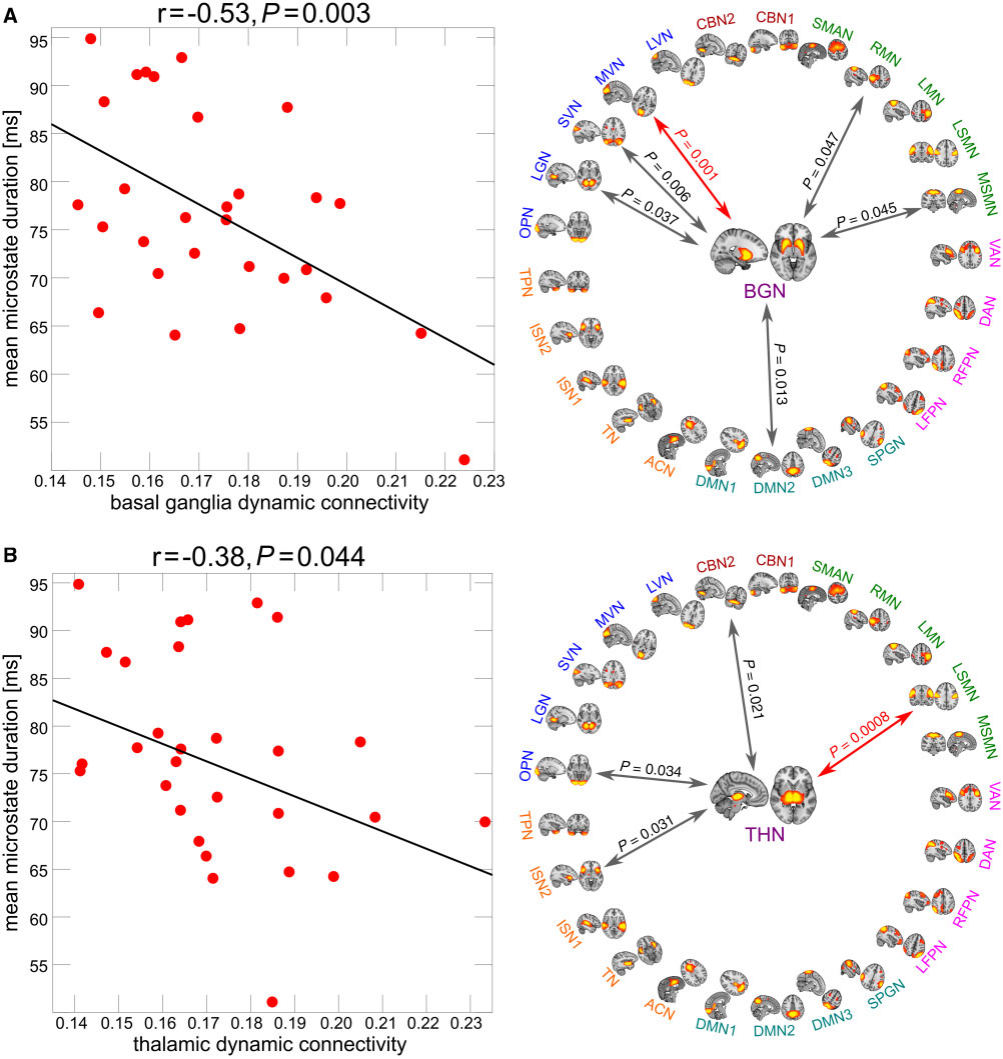
**Relation between microstate dynamics and dynamic functional MRI connectivity.** Results from Pearson’s correlation analysis between mean microstate duration and dynamic functional connectivity of (**A**) the basal ganglia network (BGN) and (**B**) the thalamic network (THN) in the Lewy body dementia group. The plots on the *right* show results from correlating mean microstate duration with each individual network connection. Grey arrows indicate significant correlations at an uncorrected threshold of *P* < 0.05 and red arrows indicate connections that survive Bonferroni correction for multiple comparisons (all correlations that are marked with an arrow were negative). All correlation coefficients and corresponding *P*-values are shown in Supplementary Tables 15 and 16 and all network names and locations can be found in Supplementary Table 14.

In the Lewy body dementia group, mean variability of connectivity between the basal ganglia network and all other networks was negatively related to mean microstate duration (r = −0.53, *P* = 0.003; Fig. 5A). When considering each connection separately, there were six networks whose dynamic interaction with the basal ganglia network was negatively correlated with mean microstate duration: two motor networks (right motor network and medial sensorimotor network), three visual networks (medial visual network, superior visual network, and lingual gyrus network) and the default mode network 2 (all *P* < 0.05, uncorrected) (Supplementary Table 15). After correcting for multiple comparisons, the dynamic interaction between the basal ganglia network and the medial visual network was still significantly correlated with mean microstate duration.

For the thalamic network, overall dynamic connectivity was also negatively related to mean microstate duration (r = −0.38, *P* = 0.044; Fig. 5B). When considering each connection separately, there were four networks whose dynamic interaction with the thalamic network was negatively correlated with mean microstate duration: the insular network 2, the lateral sensorimotor network, the occipital pole network, and the cerebellar network 2 (all *P* < 0.05, uncorrected, see Supplementary Table 16). After correcting for multiple comparisons, the dynamic interaction between the thalamic network and the lateral sensorimotor network was still significantly correlated with mean microstate duration.

Supplementary Fig. 2 shows correlations between overall dynamic connectivity of basal ganglia and thalamic networks in healthy controls and Alzheimer’s disease, none of which were significant.

## Discussion

In this study, we investigated changes in brain dynamics in Lewy body dementia compared to healthy ageing and Alzheimer’s disease using an EEG microstate analysis to assess temporal characteristics of brain activity on a subsecond timescale and the relation between microstate dynamics and large-scale functional MRI network dynamics within the cortical-basal ganglia-thalamic loop.

### Microstate dynamics

We found a marked and generalized slowing of microstate dynamics in Lewy body dementia compared to both healthy controls and Alzheimer’s disease patients while temporal microstate characteristics in Alzheimer’s disease were largely comparable to healthy control levels. Patients with Lewy body dementia stayed in the same microstate class for longer consecutive periods of time and switched less frequently between different states than healthy controls and Alzheimer’s disease patients. This was not specific to a certain microstate class as reported for other diseases (Koenig *et al.*, 1999; Kikuchi *et al.*, 2011; Nishida *et al.*, 2013), but rather a general pattern observed for all classes, which indicates that general microstate timing mechanisms are affected in Lewy body dementia.

The observed slowing of microstate dynamics in Lewy body dementia indicates a relative loss of resting state brain variability compared to healthy ageing and Alzheimer’s disease and is in line with our previous observation of a loss of brain network flexibility in dementia with Lewy bodies as evidenced by dynamic functional MRI network analysis (Schumacher *et al.*, 2018*a*). The importance of variability in the brain has been confirmed in many studies (see Garrett *et al.*, 2013 for a review) relating less variability to ageing (Guitart-Masip *et al.*, 2016; Grady and Garrett, 2018) and poorer performance on various cognitive tests (McIntosh *et al.*, 2008; Jia *et al.*, 2014). Reduced microstate dynamics in Lewy body dementia could therefore be an indicator of less flexible, and ineffective, brain functioning.

Apart from being an indicator of brain variability at rest, microstates show elaborate dynamic properties that are important for optimal brain functioning. In the healthy brain, microstate sequences have been shown to exhibit scale-free or fractal dynamics, i.e. the microstate time course is statistically self-similar across multiple timescales (Van De Ville *et al.*, 2010). The observation of scale-free properties in a dynamic system indicates that the system operates near a point of criticality, fluctuating around a phase transition (Tagliazucchi *et al.*, 2012; Hesse and Gross, 2014). This state makes the system optimally adaptable enabling it to respond to incoming information and unpredictable stimuli by providing a self-organizing mechanism and preventing the emergence of excessive periodicity at the same time (Goldberger *et al.*, 2002). The extent of scale-free dynamics can also be used as an indicator of a system’s dynamic complexity with a reduction in fractal dimension indicating a loss of system complexity (Zappasodi *et al.*, 2014). In the context of microstate sequences, it was shown that scale-free properties are preserved when the temporal sequence of the microstate labels is randomized, whereas these long-range dependencies are lost when equalizing microstate duration (Van De Ville *et al.*, 2010). This shows that the exact sequence of microstate classes is not crucial, but rather their duration seems to be the key parameter for the emergence of scale-free dynamics and thus optimal network properties. The observed abnormalities in microstate timing in Lewy body dementia, we would therefore argue, could have significant consequences for the functioning of the whole brain network by potentially disturbing its intricate fractal dynamics. However, further work is required to assess the effect of the observed increase in microstate duration in Lewy body dementia on the fractality of the dynamic system more directly.

We found a correlation between the severity of cognitive fluctuations and temporal microstate abnormalities in the dementia with Lewy bodies group suggesting that more severe cognitive fluctuations are related to a greater slowing of microstate dynamics; this relationship was stronger for the cognitive/attentional dimensions of cognitive fluctuations as opposed to arousal or alertness (Bliwise *et al.*, 2014). Additionally, we observed largely intact microstate dynamics in an Alzheimer’s disease group of comparable dementia severity. This indicates that the alterations in dynamic properties in Lewy body dementia might drive the brain network away from the point of criticality that is important for healthy cognitive functioning towards a state that allows for the emergence of cognitive symptoms that are specific to Lewy body dementia such as fluctuating cognition (Ferman *et al.*, 2004). However, the relationship between microstate dynamics and the severity of cognitive fluctuations was specific to the dementia with Lewy bodies group and was not observed in the Parkinson’s disease dementia patients. This might suggest a different aetiology of cognitive fluctuations in these patients even though clinically they present very similarly (Ballard *et al.*, 2002*a**,**b*; Varanese *et al.*, 2010). Some of this may also relate to difficulties in assessing fluctuating cognition in patients with more advanced Parkinson’s disease due to the confounding presence of motor fluctuations or the more significant levodopa load in these patients, although notably we did not see any association between LEDD and microstate metrics.

The observation of largely preserved microstate dynamics in Alzheimer’s disease agrees with two previous studies that similarly reported no differences between Alzheimer’s disease patients and age-matched controls in terms of the microstates’ temporal characteristics (Nishida *et al.*, 2013; Grieder *et al.*, 2016). In contrast to our results, Nishida *et al.* (2013) found that transition probabilities in Alzheimer’s disease patients showed a pattern that was compatible with random transitions. Alzheimer’s patients in this previous study showed a comparable level of cognitive impairment to our patients. However, patients in the Nishida *et al.* (2013) study were not taking any cholinergic medications whereas the large majority of our patients were on cholinesterase inhibitors, which have been shown to alter resting state EEG characteristics in Alzheimer’s disease (Babiloni *et al.*, 2013) and might thus be an explanation for the different results.

An alteration in the topographical structure of the microstates was only observed in Alzheimer’s disease while topographies in Lewy body dementia did not differ significantly from healthy controls. This highlights again that it is primarily microstate dynamics that seem to be affected by Lewy body dementia. In contrast, the change in microstate topographies in Alzheimer’s disease might be due to the greater structural abnormalities in this condition compared to Lewy body dementia (Mak *et al.*, 2015*a*, *b*).

With respect to previous EEG studies in Lewy body dementia, a general slowing of oscillatory EEG activity as evidenced by a slowing of the dominant frequency in posterior regions is a well-established finding (Cromarty *et al.*, 2015; Bonanni *et al.*, 2016; Peraza *et al.*, 2018; Stylianou *et al.*, 2018) and thus it could be argued that this global change is driving our observed group differences in microstate dynamics. However, when testing the correlation between dominant frequency and mean microstate duration in the Lewy body dementia group, we only found a weak negative correlation, which was not statistically significant. This indicates that while generalized EEG slowing might partially contribute to microstate slowing, it does not fully explain the relative loss of microstate dynamics in Lewy body dementia. In contrast, the number of GFP peaks per second was positively correlated with dominant frequency in the Lewy body dementia group indicating that the group differences in the number of GFP peaks per second were influenced by differences in dominant frequency between the groups (Lehmann *et al.*, 1987; Peraza *et al.*, 2018). However, we showed that, overall, our results can be replicated when fitting the microstates on all data instead of the GFP peaks, further suggesting that the well established finding of EEG slowing in Lewy body dementia is not equivalent to the slowing of microstate dynamics that we describe here. Nevertheless, the frequency analysis indicated that there is a potential inter-relation between a shift of EEG power from higher to lower frequencies and a slowing of microstate dynamics as previously suggested by Koenig *et al.* (2002). Further work is therefore required to understand the exact relationship between microstate slowing and general EEG slowing in Lewy body dementia.

### Origins of microstate disturbances in Lewy body dementia

Even though previous studies have found a link between the rapidly changing EEG microstate sequences and slower changes of the functional MRI signal (Britz *et al.*, 2010; Musso *et al.*, 2010; Custo *et al.*, 2017), it remains largely unknown which processes in the brain might be responsible for the emergence of the precise microstate timing and hence their complex dynamic properties (Michel and Koenig, 2017). In our study, we found an association between less dynamic connectivity between basal ganglia and thalamic networks with large-scale cortical networks and a loss of microstate dynamics in Lewy body dementia. These findings provide, for the first time, evidence to suggest that the dynamic interaction within the cortical-basal ganglia-thalamic loop plays a part in the modulation of global microstate dynamics. This is relevant from a Lewy body dementia perspective as thalamic and basal ganglia dysfunction is a hallmark of Lewy body diseases (Pimlott *et al.*, 2006; McKeith *et al.*, 2007; Delli Pizzi 2015*a*, *b*; Watson *et al.*, 2017). Our results therefore support the conjecture that key subcortical abnormalities have broader impacts on the overall functioning of the whole-brain network in Lewy body dementia: we speculate that structural and functional changes within subcortical structures associated with Lewy body disease contribute to an impairment in the dynamic interaction between these subcortical and large-scale cortical networks. This in turn might lead to the loss of crucial dynamic properties and hence a reduction in brain adaptability and efficiency as described above. Additionally, these results provide a possible explanation for how strategic pathology in subcortical structures in Lewy body dementia can have more widespread impact on cognitive functions and symptom manifestation, especially with respect to cognitive fluctuations (Delli Pizzi *et al.*, 2015*a*).

Apart from being relevant to our understanding of brain abnormalities in Lewy body dementia, the present study might also help to further our more general understanding of microstate dynamics by providing a first hint at how dynamic microstate properties might be modulated by subcortical-cortical dynamics. This has wider implications for a better mechanistic understanding of other diseases that are characterized by microstate abnormalities such as schizophrenia and depression (Strik *et al.*, 1995; Koenig *et al.*, 1999; Lehmann *et al.*, 2005).

### Limitations

Our study has some limitations. First, most of the patients were taking cholinesterase inhibitors and/or dopaminergic medication which can influence functional MRI and EEG signals (Babiloni *et al.*, 2013; Szewczyk-Krolikowski *et al.*, 2014). Regarding the use of dopaminergic medication we confirmed that there were no differences in microstate temporal characteristics between patients ON and OFF medication and no relation to LEDD (Supplementary Table 9). However, with respect to the use of cholinesterase inhibitors, such a comparison was not possible due to the very small number of patients in the latter group and this therefore remains a limitation. More broadly this is relevant, given *a priori* evidence demonstrating a relationship between disruption to the cholinergic system and cognitive fluctuations (Ballard *et al.*, 2002*a**,**b*; Pimlott *et al.*, 2006; Colloby *et al.*, 2017) as well as remediation of this symptom with cholinesterase inhibitor treatment in Lewy body dementia (Onofrj *et al.*, 2003). The intimate role of cholinergic efferents, for example, from the pedunculopontine nucleus in regulating cortico-thalamic outflow may therefore apposite in shaping microstate dynamics and contribute to our observations. Further work will be required to unpick this conjecture.

In addition, we used non-concurrent EEG-functional MRI recordings in our study and thus we can only draw limited conclusions with respect to a causal influence of network dynamics on microstate characteristics. While the present results provide an indication of a link between functional MRI and EEG dynamics, studying concurrent EEG-functional MRI data in the future will allow us to draw more concrete conclusions, especially with respect to the causal relation between microstate characteristics and large-scale network dynamics.

## Conclusions

We report a profound slowing of microstate dynamics in Lewy body dementia that clearly distinguished this form of dementia from Alzheimer’s disease and healthy ageing and which was related to the severity of cognitive fluctuations in the dementia with Lewy bodies patients. Disturbances to the precise timing of microstate sequences in Lewy body dementia may lead to a breakdown of the fractal properties of the system therefore causing a loss of complexity and adaptability of the brain network that is crucial for its healthy functioning and which may in turn be related to the emergence of transient clinical symptoms such as cognitive fluctuations. Additionally, by using Lewy body dementia as a probe pathology we found a potential link between large-scale network fluctuations and microstate dynamics, suggesting that dynamic interactions within the cortical-basal ganglia-thalamic loop might play a role in the modulation of EEG dynamics.

## Funding

J.S. is supported by the Alzheimer’s Society Doctoral Training Centre at Newcastle University. M.K. is supported by the Engineering and Physical Sciences Research Council of the United Kingdom Grant EP/K026992/1. The research was supported by a Wellcome Trust Intermediate Clinical Fellowship (WT088441MA) to J.-P.T., Northumberland Tyne and Wear NHS Foundation Trust, by National Institute for Health Research (NIHR) Newcastle Biomedical Research Centre (BRC) based at Newcastle upon Tyne Hospitals NHS Foundation Trust and Newcastle University, and by Alzheimer’s Research UK.

## Competing interests

The authors report no competing interests.

## Supplementary Material

awz069_Supplementary_Material

## Abbreviation

GFP: global field power
