# Supplementary material for: Dysfunctional brain dynamics and their origin in Lewy body dementia
Source: Brain. 2019 Apr 1;142(6):1767–82. doi: 10.1093/brain/awz069 (PMC6536851; doi:10.1093/brain/awz069)
Supplement: awz069_Supplementary_Material [file awz069_supplementary_material.pdf]

Supplementary Material: Dysfunctional brain dynamics and their origin in Lewy body dementia

Julia Schumacher[a], Luis R. Peraza[a,b], Michael Firbank[a], Alan J. Thomas[a], Marcus Kaiser[b,c], Peter Gallagher[c], John T. O'Brien[d], Andrew M. Blamire[e], John-Paul Taylor[a]

Correspondence: Julia Schumacher (j.a.schumacher2@newcastle.ac.uk)

Contents

1. Comparison of Lewy body dementia subgroups 2
2. Group comparison of microstate characteristics with matched dementia groups 4
3. Group comparison of microstate duration and occurrence including GEV covariate 8
4. TANOVA results 9
5. Post-hoc analysis for microstate coverage 10
6. Effect of dopaminergic medication on microstate characteristics 11
7. Microstate fitting on all data 12
8. Clinical correlations 13
9. Analysis of transition probabilities 15
10. Frequency analysis 16
11. Dynamic connectivity analysis: Methods 17
12. Dynamic connectivity analysis: Results 19
13. Supplementary references 25

1. Comparison of Lewy body dementia subgroups

Table S1: Demographic and clinical comparison of DLB and PDD subgroups.

	DLB (N=25)	PDD (N=17)	Between-group differences
Male: female	20:5	16:1	χ^2=1.65, p=0.20[a]
Age	76.2 (6.2)	72.8 (6.2)	t_{40}=1.71, p=0.10[c]
AChEI	23	13	χ^2 =2.00, p=0.16[a]
PD meds	12	17	χ^2 =12.80, p<0.001[a]
Duration	3.5 (2.3)	2.8 (1.5)[e]	U =174, p=0.48[b]
MMSE	22.7 (4.3)	23.8 (2.6)	t_{40} =0.92, p =0.36[c]
CAMCOG	74.8 (12.8)	77.1 (8.2)	t_{40} =0.63, p =0.53[c]
UPDRS	16.2 (7.5)	26.6 (5.5)	t_{40} =4.88, p<0.001[c]
CAF total	4.1 (4.1)[d]	6.3 (4.4)[e]	t_{38} =1.55, p=0.13[c]
Mayo total	13.3 (5.9)[d]	14.9 (5.4)[e]	t_{38}=0.88, p=0.39[c]
Mayo cogn	2.5 (1.8)[d]	3.2 (1.9)[e]	t_{38}=1.10, p=0.28[c]
NPI total	10.2 (6.3)[d]	20.1 (12.6)	t_{39}=3.31, p=0.002[c]
NPI hall	1.7 (1.9)[d]	2.2 (2.1)	t_{39}=0.85, p=0.40[c]

AChEI, number of patients taking acetylcholinesterase inhibitors; CAF total, Clinical Assessment of Fluctuations total score; CAMCOG, Cambridge Cognitive Examination; DLB, Dementia with Lewy bodies; Duration, duration of cognitive symptoms in years; Mayo total, Mayo Fluctuations Scale; Mayo cognitive, Mayo Fluctuation cognitive subscale; Mayo arousal, Mayo Fluctuations arousal subscale; MMSE, Mini Mental State Examination; PDD, Parkinson's disease dementia; PD meds, number of patients taking dopaminergic medication; UPDRS, Unified Parkinson's Disease Rating Scale; NPI, Neuropsychiatric Inventory; NPI hall, NPI hallucination subscore

[a] Chi-square test DLB, PDD; [b] Mann Whitney U test DLB, PDD; [c] Student's t-test DLB, PDD.
[d] N=24, [e] N=16

Table S2: Mean [95% confidence interval] of microstate duration and microstate occurrence per second in the DLB and PDD subgroups and results from group comparison using two-sample t-tests.

	DLB	PDD	t-test
duration			
mean	78.53	74.86	t(40)=1.2
	[74.9,82.2]	[69.1,80.6]	p=0.24
A	71.00	70.93	t(40)=0.02
	[66.9,75.1]	[65.7,76.2]	p=0.98
B	71.11	70.77	t(40)=0.1
	[67.2,75.0]	[64.1,77.5]	p=0.92
C	76.98	73.89	t(40)=0.9
	[72.2,81.7]	[68.3,79.4]	p=0.39
D	83.58	74.92	t(40)=2.0
	[78.0,89.2]	[67.9,82.0]	p=0.05
E	79.2	74.26	t(40)=1.0
	[72.3,86.2]	[67.8,80.7]	p=0.30
occurrence			
mean	13.25	13.95	t(40)=1.2
	[12.7,13.8]	[12.8,15.1]	p=0.22
A	2.47	2.76	t(40)=1.6
	[2.2,2.7]	[2.4,3.1]	p=0.13
B	2.40	2.67	t(40)=2.0
	[2.3,2.5]	[2.4,2.9]	p=0.053
C	2.63	2.86	t(40)=1.5
	[2.5,2.8]	[2.6,3.2]	p=0.14
D	3.03	2.83	t(40)=1.0
	[2.8,3.2]	[2.5,3.2]	p=0.32
E	2.72	2.83	t(40)=0.9
	[2.6,2.9]	[2.6,3.1]	p=0.38

DLB, dementia with Lewy bodies; PDD, Parkinson’s disease dementia

2. Group comparison of microstate characteristics with matched dementia groups

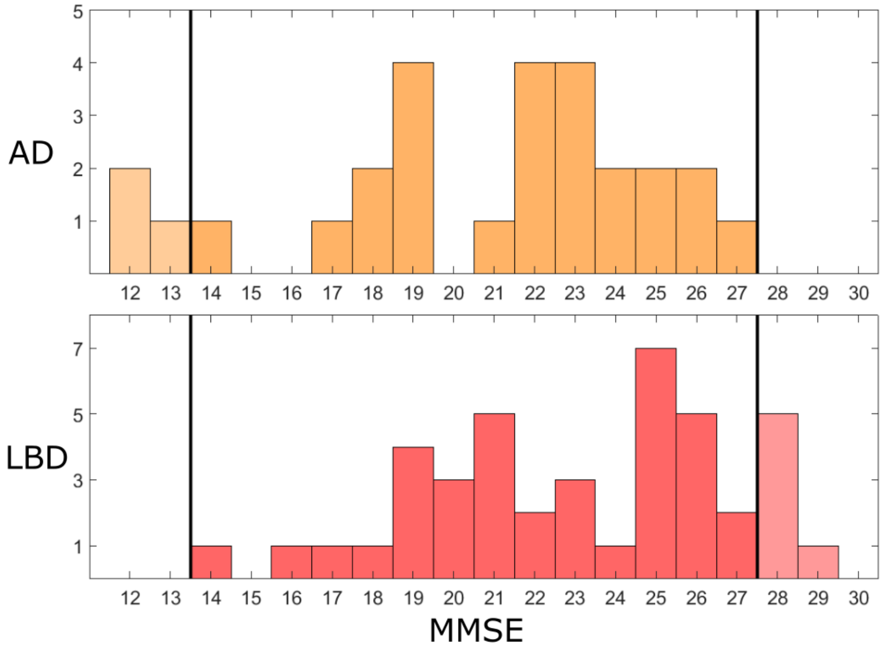

Figure S1: Selection of matched dementia groups by excluding three Alzheimer's disease patients with MMSE<14 and six Lewy body dementia (five dementia with Lewy bodies and one Parkinson's disease dementia) patients with MMSE>27.

Table S3: Demographic and clinical comparison of matched Alzheimer's disease and Lewy body dementia subgroups.

	AD (N=24)	LBD (N=36)	Between-group differences
Male: female	18:6	31:5	χ^2=1.19, p=0.28[a]
Age	74.9 (6.9)	74.6 (6.7)	t_{58}=0.20, p=0.84[c]
AChEI	22[d]	32[e]	χ^2=0.39, p =0.54[a]
PD meds	1[d]	24[e]	χ^2=23.24, p<0.001[a]
Duration	3.8 (2.2)[d]	3.3 (2.1)[e]	U=322, p=0.19[b]
MMSE	21.7 (3.3)	22.3 (3.3)	t_{58}=0.65, p=0.52[c]
CAMCOG	71.5 (10.9)	73.2 (9.7)	t_{58}=0.62, p=0.54[c]
UPDRS	1.8 (1.6)	20.9 (8.5)	t_{58}=10.82, p<0.001[c]
CAF total	0.3 (0.7)[d]	5.4 (4.5)[e]	t_{55}=5.46, p<0.001[c]
Mayo total	8.4 (4.0)[d]	15.2 (4.8)[e]	t_{55}=5.62, p<0.001[c]
Mayo cogn	1.7 (1.9)[d]	3.2 (1.7)[e]	t_{55}=3.19, p=0.002[c]
NPI total	7.0 (6.8)[d]	15.5 (10.7)[f]	t_{56}=3.42, p=0.001[c]
NPI hall	0.04 (0.2)[d]	2.0 (2.0)[f]	t_{56}=4.66, p<0.001[c]

AChEI, number of patients taking acetylcholinesterase inhibitors; AD, Alzheimer's disease; CAF total, Clinical Assessment of Fluctuations total score; CAMCOG, Cambridge Cognitive Examination; Duration, duration of cognitive symptoms in years; HC, healthy controls; LBD, Lewy body dementia; Mayo total, Mayo Fluctuations Scale; Mayo cognitive, Mayo Fluctuation cognitive subscale; Mayo arousal, Mayo Fluctuations arousal subscale; MMSE, Mini Mental State Examination; PD meds, number of patients taking dopaminergic medication; UPDRS, Unified Parkinson's Disease Rating Scale; NPI, Neuropsychiatric Inventory; NPI hall, NPI hallucination subscore

[a] Chi-square test AD, LBD; [b] Mann Whitney U test AD, LBD; [c] Student's t-test AD, LBD.

[d] N=23, [e] N=34, [f] N=35

Table S4: Mean microstate duration [95% confidence intervals] for microstate classes A to E with matched dementia groups (see Supplementary Fig. S1 and Supplementary Table S3), and results from group comparison using univariate ANOVAs and pairwise post-hoc tests. Post-hoc p-values are Bonferroni-corrected for multiple comparisons.

	HC	AD	LBD	ANOVA	post-hoc (p-value)		
					HC-AD	HC-LBD	AD-LBD
mean	64.7 [60.1,69.2]	65.6 [61.7,69.6]	77.6 [74.4,80.9]	F(2,75)=16.0, p<0.001	1.0	<0.001	<0.001
A	56.6 [52.1,61.1]	64.7 [60.8,68.7]	71.9 [68.7,75.2]	F(2,75)=15.5 p<0.001	0.03	<0.001	0.02
B	57.6 [52.9,62.3]	61.0 [56.9,65.1]	71.6 [68.2,74.9]	F(2,75)=14.4 p<0.001	0.86	<0.001	<0.001
C	60.8 [56.1,65.5]	66.2 [62.1,70.3]	76.4 [73.0,79.7]	F(2,75)=16.6 p<0.001	0.26	<0.001	0.001
D	64.2 [57.7,70.7]	64.7 [59.1,70.3]	79.7 [75.1,84.3]	F(2,75)=11.8 p<0.001	1.0	0.001	<0.001
E	67.6 [60.9,74.3]	65.0 [59.2,70.9]	78.4 [73.6,83.1]	F(2,75)=7.2 p=0.001	1.0	0.03	0.002

AD, Alzheimer's disease; ANOVA, analysis of variance; HC, healthy controls; LBD, Lewy body dementia

Table S5: Mean microstate occurrence per second [95% confidence intervals] for microstate classes A to E for matched dementia groups (see Supplementary Fig. S1 and Supplementary Table S3), and results from group comparison using univariate ANOVAs and pairwise post-hoc tests. Post-hoc p-values are Bonferroni-corrected for multiple comparisons.

	HC	AD	LBD	ANOVA	post-hoc (p-value)		
					HC-AD	HC-LBD	AD-LBD
mean	16.1 [15.2,17.0]	15.8 [15.0,16.6]	13.5 [12.8,14.1]	F(2,75)=15.1 p<0.001	1.0	<0.001	<0.001
A	3.0 [2.6,3.3]	3.2 [2.9,3.5]	2.6 [2.3,2.8]	F(2,75)=4.7 p=0.01	1.0	0.24	0.01
B	3.0 [2.8,3.3]	2.9 [2.7,3.1]	2.5 [2.3,2.7]	F(2,75)=6.9 p=0.002	1.0	0.003	0.03
C	3.1 [2.9,3.5]	3.4 [3.1,3.6]	2.7 [2.5,2.9]	F(2,75)=9.3 p<0.001	0.76	0.04	<0.001
D	3.4 [3.2,3.7]	3.3 [3.0,3.5]	2.9 [2.7,3.1]	F(2,75)=6.1 p=0.004	1.0	0.006	0.05
E	3.5 [3.2,3.8]	3.1 [2.9,3.3]	2.7 [2.6,2.9]	F(2,75)=10.5 p<0.001	0.08	<0.001	0.08

AD, Alzheimer’s disease; ANOVA, analysis of variance; HC, healthy controls; LBD, Lewy body dementia

3. Group comparison of microstate duration and occurrence including GEV covariate

There was a trend for a group difference in global explained variance (GEV) of five microstate classes (univariate ANOVA, $F(2,84)=3.01$, $p=0.06$). Post-hoc tests (Bonferroni-corrected) showed that there were no differences between Alzheimer's disease and controls ($p=0.20$) or between Lewy body dementia and controls ($p=1.0$). However, there was a trend for smaller GEV in Alzheimer's disease compared to Lewy body dementia ($p=0.07$). To test whether these marginal group differences in GEV had an effect on the results from the group comparison of microstate characteristics, the analyses reported in Section 3.4. of the main text were repeated including GEV as covariate. There was a group difference for mean microstate duration ($F(2,83)=17.51$, $p<0.001$) and microstate occurrence ($F(2,83)=17.14$, $p<0.001$). Post-hoc tests revealed that microstate duration was increased in Lewy body dementia compared to controls ($p<0.001$) and Alzheimer's disease ($p=0.001$) with no difference between Alzheimer's disease and controls ($p=0.13$). Microstate occurrence per second was reduced in Lewy body dementia compared to controls ($p<0.001$) and Alzheimer's disease ($p=0.003$) with no significant difference between Alzheimer's disease and controls ($p=0.07$). Thus, including GEV as a covariate did not change the overall significance of the results.

4. TANOVA results

Table S6: P-values from TANOVA test of microstate topographies for microstate classes A-E between groups. The overall two-way TANOVA with microstate class as within-subject factor and group as between-subject factor resulted in a main effect of group ($p<0.001$), a main effect of microstate class ($p<0.001$), but no interaction between the two factors ($p=0.45$).

	all groups	HC-AD	HC-LBD	AD-LBD
A	<0.001	<0.001	0.15	<0.001
B	<0.001	<0.001	0.36	<0.001
C	0.014	0.021	0.38	0.009
D	0.036	0.049	0.40	0.027
E	0.006	0.048	0.37	<0.001

AD, Alzheimer's disease; HC, healthy controls; LBD, Lewy body dementia

Table S7: P-values from TANOVA test of microstate topographies for microstate classes A to E with matched dementia groups (see Supplementary Figure S1 and Supplementary Table S3). The overall two-way TANOVA with microstate class as within-subject factor and group as between-subject factor resulted in a main effect of group ($p<0.001$), a main effect of microstate class ($p<0.001$), but no interaction between the two factors ($p=0.47$).

	all groups	HC-AD	HC-LBD	AD-LBD
A	<0.001	<0.001	0.11	<0.001
B	<0.001	<0.001	0.22	<0.001
C	0.013	0.017	0.45	0.009
D	0.039	0.034	0.47	0.031
E	0.004	0.027	0.46	<0.001

AD, Alzheimer's disease; HC, healthy controls; LBD, Lewy body dementia

5. Post-hoc analysis for microstate coverage

Table S8: Mean microstate coverage [95% confidence intervals] for microstate classes A to E and the three clinical groups, and results from group comparison using univariate ANOVAs and pairwise post-hoc tests. Post-hoc p-values are Bonferroni-corrected for multiple comparisons.

	HC	AD	LBD	ANOVA	post-hoc (p-value)		
					HC-AD	HC-LBD	AD-LBD
A	0.18 [0.15,0.20]	0.20 [0.19,0.22]	0.18 [0.17,0.20]	F(2,84)=2.15 p=0.12	0.20	1.0	0.28
B	0.18 [0.16,0.19]	0.18 [0.16,0.19]	0.18 [0.17,0.19]	F(2,84)=0.04 p=0.96	1.0	1.0	1.0
C	0.19 [0.17,0.21]	0.22 [0.20,0.23]	0.20 [0.19,0.21]	F(2,84)=2.12 p=0.13	0.14	0.99	0.53
D	0.22 [0.20,0.25]	0.21 [0.19,0.23]	0.23 [0.21,0.25]	F(2,84)=1.60 p=0.21	1.0	1.0	0.23
E	0.24 [0.21,0.26]	0.20 [0.18,0.22]	0.21 [0.19,0.23]	F(2,84)=3.09 p=0.051	0.049	0.18	1.0

AD, Alzheimer's disease; ANOVA, analysis of variance; HC, healthy controls; LBD, Lewy body dementia

6. Effect of dopaminergic medication on microstate characteristics

Table S9: Mean [95% confidence interval] of microstate duration and microstate occurrence per second comparing Lewy body dementia (LBD) patients who were not on dopaminergic medication (no PD meds, N=13) to those Lewy body dementia patients who are taking dopaminergic mediation (PD meds, N=29). There was no significant correlation between levodopa equivalent daily dose (Tomlinson *et al.*, 2010) and mean microstate duration (Pearson's r=0.02, p=0.92) or mean microstate occurrence per second (Pearson's r=-0.01, p=0.94).

	LBD,<br>no PD meds	LBD,<br>PD meds	t-test
duration			
mean	80.3<br>[75.1,85.6]	75.6<br>[71.7,79.5]	t(40)=1.5<br>p=0.16
A	72.9<br>[66.9,78.9]	70.1<br>[66.3,73.9]	t(40)=0.83<br>p=0.41
B	73.2<br>[67.3,79.0]	70.0<br>[65.7,74.3]	t(40)=0.88<br>p=0.38
C	79.6<br>[74.1,85.0]	74.0<br>[69.5,78.5]	t(40)=1.5<br>p=0.14
D	82.1<br>[72.7,91.6]	79.1<br>[74.0,84.3]	t(40)=0.63<br>p=0.53
E	81.9<br>[70.2,93.5]	75.1<br>[70.2,80.1]	t(40)=1.3<br>p=0.19
occurrence			
mean	12.9<br>[12.2,13.7]	13.8<br>[13.0,14.5]	t(40)=1.4<br>p=0.16
A	2.5<br>[2.1,2.8]	2.6<br>[2.4,2.9]	t(40)=0.91<br>p=0.37
B	2.3<br>[2.2,2.5]	2.6<br>[2.4,2.8]	t(40)=1.7<br>p=0.10
C	2.6<br>[2.3,2.8]	2.8<br>[2.6,3.0]	t(40)=1.3<br>p=0.19
D	2.9<br>[2.6,3.1]	3.0<br>[2.7,3.2]	t(40)=0.46<br>p=0.65
E	2.7<br>[2.4,3.0]	2.8<br>[2.6,3.0]	t(40)=0.84<br>p=0.41

7. Microstate fitting on all data

To test whether group differences in microstate duration and occurrence were merely due to group differences in the number of global field power (GFP) peaks per second, we repeated the microstate analysis, but fitting group microstates to each time point of the individual subject data instead of only fitting to data at GFP peaks. This analysis was performed in Cartool using default smoothing parameters (smoothing half window size of 3 time frames, smoothing strength $\lambda=10$, and rejecting small segments below 3 time frames).

Subsequently, microstate characteristics were computed in the same way as described in the main text removing possibly truncated microstates from the epoch boundaries. Mean microstate duration and occurrence were compared between the groups using univariate ANOVAs. There was an overall group effect for microstate duration ($F(2,84)=38.66$, $p<0.001$). Post-hoc tests with Bonferroni correction for multiple comparisons revealed that microstate duration was longer in the Lewy body dementia group compared to both controls ($p<0.001$) and Alzheimer's disease ($p<0.001$) whereas there was no significant difference between controls and Alzheimer's disease ($p=0.40$).

There was also an overall group effect for microstate occurrence ($F(2,84)=50.26$, $p<0.001$). Post-hoc tests showed that microstate occurrence per second was lower in Lewy body dementia compared to controls ($p<0.001$) and Alzheimer's disease ($p<0.001$) with no significant difference between controls and Alzheimer's disease ($p=0.07$).

8. Clinical correlations

Table S10: Spearman's correlation between mean microstate duration and additional fluctuation scores (CAF) and global cognitive scores (MMSE and CAMCOG). P-values are uncorrected for multiple comparisons.

Fluctuation scores			
	LBD	DLB	PDD
Mayo total	0.36 (p=0.023)	0.56 (p=0.004)	0.07 (p=0.79)
Mayo cognitive	0.33 (p=0.035)	0.51 (p=0.012)	0.17 (p=0.54)
Mayo arousal	0.27 (p=0.10)	0.45 (p=0.027)	0.04 (p=0.88)
CAF total	0.24 (p=0.14)	0.34 (p=0.10)	0.18 (p=0.50)
CAF duration	0.23 (p=0.16)	0.38 (p=0.07)	0.06 (p=0.82)
CAF frequency	0.22 (p=0.17)	0.20 (p=0.34)	0.47 (0.06)
Global cognitive scores			
	all dementia patients	AD	LBD
MMSE	-0.09 (p=0.47)	-0.28 (p=0.17)	-0.29 (p=0.06)
CAMCOG	-0.005 (p=0.97)	-0.42 (p=0.03)	-0.02 (p=0.91)

AD, Alzheimer's disease; CAF total, Clinical Assessment of Fluctuations total score; CAMCOG, Cambridge Cognitive Examination; DLB, Dementia with Lewy bodies; LBD, Lewy body dementia; Mayo total, Mayo Fluctuations Scale; Mayo cognitive, Mayo Fluctuation cognitive subscale; Mayo arousal, Mayo Fluctuations arousal; MMSE, Mini Mental State Examination; PDD, Parkinson's disease dementia.

Table S11: Spearman's correlations between Mayo fluctuation scores and microstate duration for each microstate class separately in the Lewy body dementia groups. P-values are uncorrected for multiple comparisons.

	Mayo total	Mayo cognitive	Mayo arousal
LBD			
Microstate A	0.35 (p=0.03)	0.31 (p=0.05)	0.18 (p=0.27)
Microstate B	0.31 (p=0.05)	0.31 (p=0.05)	0.11 (p=0.50)
Microstate C	0.42 (p=0.007)	0.31 (p=0.05)	0.31 (p=0.05)
Microstate D	0.16 (p=0.33)	-0.02 (p=0.91)	0.30 (p=0.07)
Microstate E	0.44 (p=0.005)	0.42 (p=0.007)	0.35 (p=0.03)
DLB			
Microstate A	0.34 (p=0.11)	0.26 (p=0.23)	0.12 (p=0.58)
Microstate B	0.52 (p=0.009)	0.42 (p=0.04)	0.25 (p=0.25)
Microstate C	0.49 (p=0.02)	0.44 (p=0.03)	0.33 (p=0.12)
Microstate D	0.35 (p=0.09)	0.16 (p=0.47)	0.48 (p=0.02)
Microstate E	0.64 (p=0.001)	0.55 (p=0.005)	0.58 (p=0.003)
PDD			
Microstate A	0.31 (p=0.24)	0.35 (p=0.19)	0.25 (p=0.36)
Microstate B	0.08 (p=0.76)	0.25 (p=0.35)	-0.08 (p=0.78)
Microstate C	0.24 (p=0.37)	0.16 (p=0.57)	0.27 (p=0.31)
Microstate D	-0.04 (p=0.87)	-0.08 (p=0.78)	0.16 (p=0.56)
Microstate E	0.12 (p=0.65)	0.31 (p=0.24)	-0.01 (p=0.96)

DLB, Dementia with Lewy bodies; Mayo total, Mayo Fluctuations Scale; Mayo cognitive, Mayo Fluctuation cognitive subscale; Mayo arousal, Mayo Fluctuations arousal; PDD, Parkinson's disease dementia.

9. Analysis of transition probabilities

Transition probabilities between different microstate classes were assessed by counting the number of transitions from each microstate class to any other class and normalising by all between-class transitions for each subject separately. If the transition from one microstate class to the next occurred randomly, i.e. irrespective of the class of the preceding microstate, transition probabilities would be proportional to the relative occurrence of the microstate classes. Under the null hypothesis of random transitions between microstates, the expected transition probability for transitions from microstate class X to class Y is therefore given by (Lehmann *et al.*, 2005):

$$P_{X \to Y}^{exp} = \frac{occurrence_X \times occurrence_Y}{1 - occurrence_X}$$

To assess the randomness of transition probabilities, a non-parametric randomisation test was applied as described in Lehmann *et al.* (2005). Within each group, the observed and expected transition probabilities were averaged across participants and the overall difference between observed and expected transition probabilities was calculated using the χ^2-distance (Lehmann *et al.*, 2005). Individual observed and expected transition probabilities were then randomly permuted 5000 times to obtain the distribution of χ^2-distance values under the null hypothesis of random transition probabilities. The p-value was calculated as the fraction of permutations in which the χ^2-distance was larger than the distance using non-permuted transition probabilities.

The overall randomisation test showed that transition probabilities were non-random in all three groups (controls: $p=0.011$, Alzheimer's disease: $p=0.001$, Lewy body dementia: $p=0.004$). There were, however, no group differences in the transition probabilities between different microstate classes (MANOVA, $F(38,134)=1.38$, $p=0.1$).

10. Frequency analysis

Table S12: Pearson's correlation between power in different frequency bands and mean microstate duration and the number of GFP peaks per second in the Lewy body dementia group. P-values are uncorrected for multiple comparisons.

	delta power	theta power	high theta power	alpha power	beta power
mean duration	r=0.35, p=0.02	r=0.23, p=0.14	r=0.26, p=0.09	r=-0.31, p=0.05	r=-0.58, p<0.001
number of GFP peaks/s	r=-0.60, p<0.001	r=-0.22, p=0.17	r=-0.10, p=0.53	r=0.45, p=0.003	r=0.71, p<0.001

11. Dynamic connectivity analysis: Methods

Resting state fMRI data were acquired with a gradient echo echo-planar imaging sequence with 25 contiguous axial slices, 128 volumes, anterior-posterior acquisition, in plane resolution = 2.0 x 2.0 mm, slice thickness = 6 mm, repetition time (TR) = 3000ms, echo time = 40ms, and field of view = 260 x 260 mm^2.

Data were preprocessed using FEAT version 6.0 in FSL (www.fmrib.ox.ac.uk/fsl) including motion correction with MCFLIRT, slice-timing correction, and spatial smoothing with a 6.0mm full width at half maximum Gaussian kernel. ICA-AROMA was applied to remove motion components from each participant's functional data (Pruim *et al.*, 2015). Eroded CSF and white matter masks were estimated using FAST in FSL and the mean signal inside the mask was regressed out of each participant's cleaned functional data. Functional images were co-registered to the structural images using boundary based registration in FSL, and normalized to standard MNI space using Advanced Normalization Tools (Avants *et al.*, 2011). Finally, functional data were temporally high-pass filtered with a cut-off of 150 s and resampled to a resolution of 4 x 4 x 4 mm^3.

Resting state networks (RSNs) were estimated from an independent set of 42 healthy control participants by applying group-ICA using FSL's MELODIC. A meta ICA approach was adopted to obtain reliable components (Biswal *et al.*, 2010; Poppe *et al.*, 2013) using a model order of 70 independent components (Abou Elseoud *et al.*, 2011). Meta ICA components were visually inspected with respect to their spatial maps (Kelly *et al.*, 2010) and 27 RSNs were identified as being of biological interest (Beckmann *et al.*, 2005; Agosta *et al.*, 2012) (Supplementary Table S13). Subsequently, FSL-dual regression was run with the 27 identified RSNs to obtain subject-specific time courses. These were further processed in Matlab (R2016b) using functions from the GIFT toolbox (http://mialab.mrn.org/software/gift/index.html) to remove remaining noise sources including (1) detrending to remove linear, quadratic, and cubic trends, (2) outlier detection based on AFNI's 3dDespike function (http://afni.nimh.nih.gov/afni) and interpolation of outliers using a third-order spline fit to the clean parts of the time courses, and (3) low-pass filtering using a fifth-order Butterworth filter with a cutoff frequency of 0.15 Hz.

The postprocessed dual regression time series were analyzed with a sliding window method to assess between-network dynamic connectivity (using Matlab and functions from GIFT (Allen *et al.*, 2014)). A tapered window was created by convolving a rectangle of 22TR (66s) with a Gaussian with sigma of 3TR and moved in steps of 1TR. A covariance matrix between all RSN-to-RSN pairs was estimated for each window separately. Since estimation of covariance based on short time series can be noisy, we estimated the regularized inverse covariance matrix using the graphical LASSO approach by imposing an L1-norm constraint on the inverse covariance matrix (Allen *et al.*, 2014). The L1 regularization parameter λ was optimized for each participant individually by evaluating the log-likelihood of unseen time windows from the same participant using 20-fold cross-validation. All

covariances were subsequently converted to correlation values and transformed into z-scores using Fisher r-to-z transformation. To control for the effect of possible covariates the z-scores were then residualized with respect to age, gender, and study membership using multiple linear regression (Damaraju *et al.*, 2014).

12. Dynamic connectivity analysis: Results

Table S13: Demographic and clinical variables for all participants that were included in the combined EEG-fMRI analysis, mean (standard deviation).

	HC (N=12)	AD (N=14)	LBD (N=29)	Between-group differences
Male: female	9:3	11:3	24:5	χ^2=0.34, p=0.84[a]
Age	76.4 (6.2)	75.0 (8.3)	74.5 (6.6)	F(2,52)=0.33, p=0.72[b]
AChEI	-	13	27	χ^2=0.01, p=0.98[c]
PD meds	-	1	21	χ^2=16.1, p<0.001[c]
Duration	-	3.9 (1.7)	3.4 (2.2)[g]	U=151, p=0.22[d]
MMSE	29.2 (0.8)	21.8 (4.1)	23.1 (3.5)	t_{41}=1.13, p=0.27[e]
CAMCOG	96.3 (2.9)	70.6 (16.4)	75.6 (11.4)	t_{41}=1.15, p=0.26[e]
UPDRS III	1.3 (1.5)	1.1 (1.2)	20.0 (8.3)	t_{41}=8.37, p<0.001[e]
CAF total	-	0.38 (1.12)[f]	5.1 (4.5)[g]	t_{39}=3.66, p=0.001[e]
Mayo total	-	8.9 (4.1)[f]	14.5 (5.4)[g]	t_{39}=3.31, p=0.002[e]
Mayo cogn	-	2.2 (1.9)[f]	2.9 (1.9)[g]	t_{39}=1.05, p=0.30[e]
NPI total	-	5.1 (4.3)[f]	15.2 (10.9)	t_{40}=3.23, p=0.003[e]
NPI hall	-	0 (0)[f]	1.9 (1.7)	t_{40}=3.96, p<0.001[e]

AChEI, number of patients taking acetylcholinesterase inhibitors; AD, Alzheimer's disease; CAF total, Clinical Assessment of Fluctuations total score; CAMCOG, Cambridge Cognitive Examination; Duration, duration of cognitive symptoms in years; HC, healthy controls; LBD, Lewy body dementia; Mayo total, Mayo Fluctuations Scale; Mayo cognitive, Mayo Fluctuation cognitive subscale; MMSE, Mini Mental State Examination; PD meds, number of patients taking dopaminergic medication for the management of Parkinson's disease symptoms; UPDRS III, Unified Parkinson's Disease Rating Scale III (motor subsection); NPI, Neuropsychiatric Inventory; NPI hall, NPI hallucination subscore

[a] Chi-square test HC, AD, LBD; [b] One-way ANOVA HC, AD, LBD; [c] Chi-square test AD, LBD; [d] Mann Whitney U test AD, LBD; [e] Student's t-test AD, LBD.

[f] N=13, [g] N=28, [h] N=13

Table S14: List of all resting state networks included in the analysis. Anatomical labels refer to bilateral areas if not stated otherwise. Locations of resting state networks are estimated from the Harvard-Oxford Cortical and Subcortical Structural Atlases and the Cerebellar Atlas included in the FMRIB's software library (FSL, www.fmrib.ox.ac.uk/fsl).

Network name		Brain regions
Lateral sensorimotor network	LSMN	Pre- and postcentral gyrus
Medial sensorimotor network	MSMN	Pre- and postcentral gyrus, supplementary motor area
Supplementary motor area network	SMAN	Supplementary motor area, precentral gyrus
Left motor network	LMN	Left post- and precentral gyrus
Right motor network	RMN	Right post- and precentral gyrus
Basal ganglia network	BGN	Putamen, caudate
Thalamic network	THN	Thalamus
Cerebellar network 1	CBN1	Cerebellum crus I, crus II
Cerebellar network 2	CBN2	Cerebellum V, VI
Medial visual network	MVN	Intracalcarine cortex, supracalcarine cortex, lingual gyrus
Lateral visual network	LVN	Superior lateral occipital cortex, precuneus
Occipital pole network	OPN	Occipital pole
Lingual gyrus network	LGN	Lingual gyrus, intracalcarine cortex
Superior visual network	SVN	Superior lateral occipital cortex, occipital pole
Temporal network	TN	Planum temporale, Heschl’s gyrus
Temporal pole network	TPN	Temporal pole
Insular network 1	ISN1	Insular cortex, frontal operculum cortex
Insular network 2	ISN2	Insular cortex, planum polare
Anterior cingulate network	ACN	Anterior cingulate cortex
Default mode network 1	DMN1	Precuneus, posterior cingulate cortex
Default mode network 2	DMN2	Precuneus
Default mode network 3	DMN3	Precuneus, superior lateral occipital cortex
Supramarginal gyrus network	SPGN	Supramarginal gyrus
Right fronto-parietal network	RFPN	Right superior lateral occipital cortex, right angular gyrus, right middle frontal gyrus, left superior lateral occipital cortex
Left fronto-parietal network	LFPN	Left superior lateral occipital cortex, right angular gyrus, left middle frontal gyrus, right superior lateral occipital cortex
Dorsal attention network	DAN	Superior parietal lobule, supramarginal gyrus, superior lateral occipital cortex
Ventral attention network	VAN	Middle frontal gyrus, inferior frontal gyrus

Table S15: Pearson correlation coefficients and p-values from correlation between mean microstate duration and basal ganglia network dynamic connectivity for each network separately in the Lewy body dementia group.

Network name	r	p-value, uncorrected	p-value, FDR-corrected
medial visual network	-0.57	0.001	0.029
superior visual network	-0.50	0.006	0.07
default mode network 2	-0.46	0.013	0.11
lingual gyrus network	-0.39	0.037	0.18
medial sensorimotor network	-0.38	0.045	0.18
right motor network	-0.37	0.047	0.18
ventral attention network	-0.36	0.058	0.18
right fronto-parietal network	-0.35	0.059	0.18
dorsal attention network	-0.31	0.10	0.23
cerebellar network 1	-0.31	0.10	0.23
insular network 1	-0.30	0.11	0.23
default mode network 1	-0.30	0.12	0.23
supplementary motor area network	-0.30	0.12	0.23
insular network 2	-0.24	0.20	0.34
left fronto-parietal network	-0.24	0.21	0.34
occipital pole network	-0.24	0.22	0.34
cerebellar network 2	-0.22	0.26	0.39
anterior cingulate network	0.19	0.32	0.45
default mode network 3	0.13	0.49	0.65
temporal pole network	-0.11	0.56	0.70
lateral sensorimotor network	0.05	0.79	0.90
lateral visual network	0.05	0.79	0.90
temporal network	0.04	0.84	0.91
supramarginal gyrus network	-0.02	0.90	0.94
left motor network	0.002	0.99	0.99

Table S16: Pearson correlation coefficients and p-values from correlation between mean microstate duration and thalamic network dynamic connectivity for each network separately in the Lewy body dementia group.

Network name	r	p-value, uncorrected	p-value, FDR-corrected
lateral sensorimotor network	-0.59	0.0008	0.019
cerebellar network 2	-0.43	0.021	0.22
insular network 2	-0.40	0.031	0.22
occipital pole network	-0.39	0.034	0.22
ventral attention network	-0.31	0.10	0.51
cerebellar network 1	-0.26	0.17	0.58
supramarginal gyrus network	-0.25	0.20	0.58
lateral visual network	-0.24	0.21	0.58
right motor network	-0.24	0.22	0.58
insular network 1	-0.23	0.23	0.58
supplementary motor area network	-0.19	0.32	0.73
medial sensorimotor network	-0.18	0.35	0.73
left fronto-parietal network	-0.15	0.44	0.85
default mode network 3	-0.12	0.52	0.94
anterior cingulate network	-0.08	0.69	0.98
left motor network	-0.07	0.70	0.98
default mode network 1	0.05	0.78	0.98
default mode network 2	-0.05	0.80	0.98
medial visual network	0.04	0.82	0.98
temporal pole network	-0.04	0.84	0.98
lingual gyrus network	-0.02	0.90	0.98
right fronto-parietal network	-0.02	0.93	0.98
dorsal attention network	-0.01	0.96	0.98
temporal network	0.01	0.96	0.98
superior visual network	0.005	0.98	0.98

Table S17: Correlation between mean basal ganglia and thalamic dynamic connectivity and microstate duration for each microstate class separately in the LBD group.

	mean BGN dynamic connectivity	mean THN dynamic connectivity
Microstate A duration	r=-0.28 (p=0.14)	r=-0.05 (p=0.79)
Microstate B duration	r=-0.30 (p=0.11)	r=-0.19 (p=0.32)
Microstate C duration	r=-0.45 (p=0.01)	r=-0.25 (p=0.19)
Microstate D duration	r=-0.30 (p=0.11)	r=-0.17 (p=0.39)
Microstate E duration	r=-0.46 (p=0.01)	r=-0.36 (p=0.06)

BGN, basal ganglia network; LBD, Lewy body dementia; THN, thalamic network.

Table S18: Correlation between basal ganglia and thalamic dynamic connectivity (for each other network separately) and microstate duration for each microstate class separately in the LBD group, only showing correlations with an uncorrected p-value<0.05.

Microstate A duration	
BGN – SVN	r=-0.41 (p=0.03)
Microstate B duration	
BGN – DAN	r=-0.50 (p=0.006)
BGN – DMN1	r=-0.47 (p=0.01)
BGN – DMN2	r=-0.40 (p=0.03)
THN – SMAN	r=-0.46 (p=0.01)
Microstate C duration	
BGN – MVN	r=-0.65 (p=0.0002)
BGN – SVN	r=-0.50 (p=0.006)
BGN – LGN	r=-0.43 (p=0.02)
BGN – DMN2	r=-0.40 (p=0.03)
THN – LSMN	r=-0.60 ((p=0.0006)
THN – CBN2	r=-0.49 (p=0.007)
Microstate D duration	
BGN – DMN1	r=-0.53 (p=0.003)
BGN – SVN	r=-0.44 (p=0.02)
Microstate E duration	
BGN – MVN	r=-0.49 (p=0.006)
BGN – DMN2	r=-0.41 (p=0.03)
BGN – MSMN	r=-0.39 (p=0.04)
BGN – LGN	r=-0.38 (p=0.04)
THN – CBN2	r=-0.54 (p=0.003)
THN – LSMN	r=-0.48 (p=0.008)
THN – OPN	r=-0.45 (p=0.01)

BGN, basal ganglia network; CBN, cerebellar network; DAN, dorsal attention network; DMN, default mode network; LGN, lingual gyrus network; LSMN, lateral sensorimotor network; MVN, medial visual network; MSMN, medial sensorimotor network; OPN, occipital pole network; SMAN, supplementary motor area network; SVN, superior visual network; THN, thalamic network.

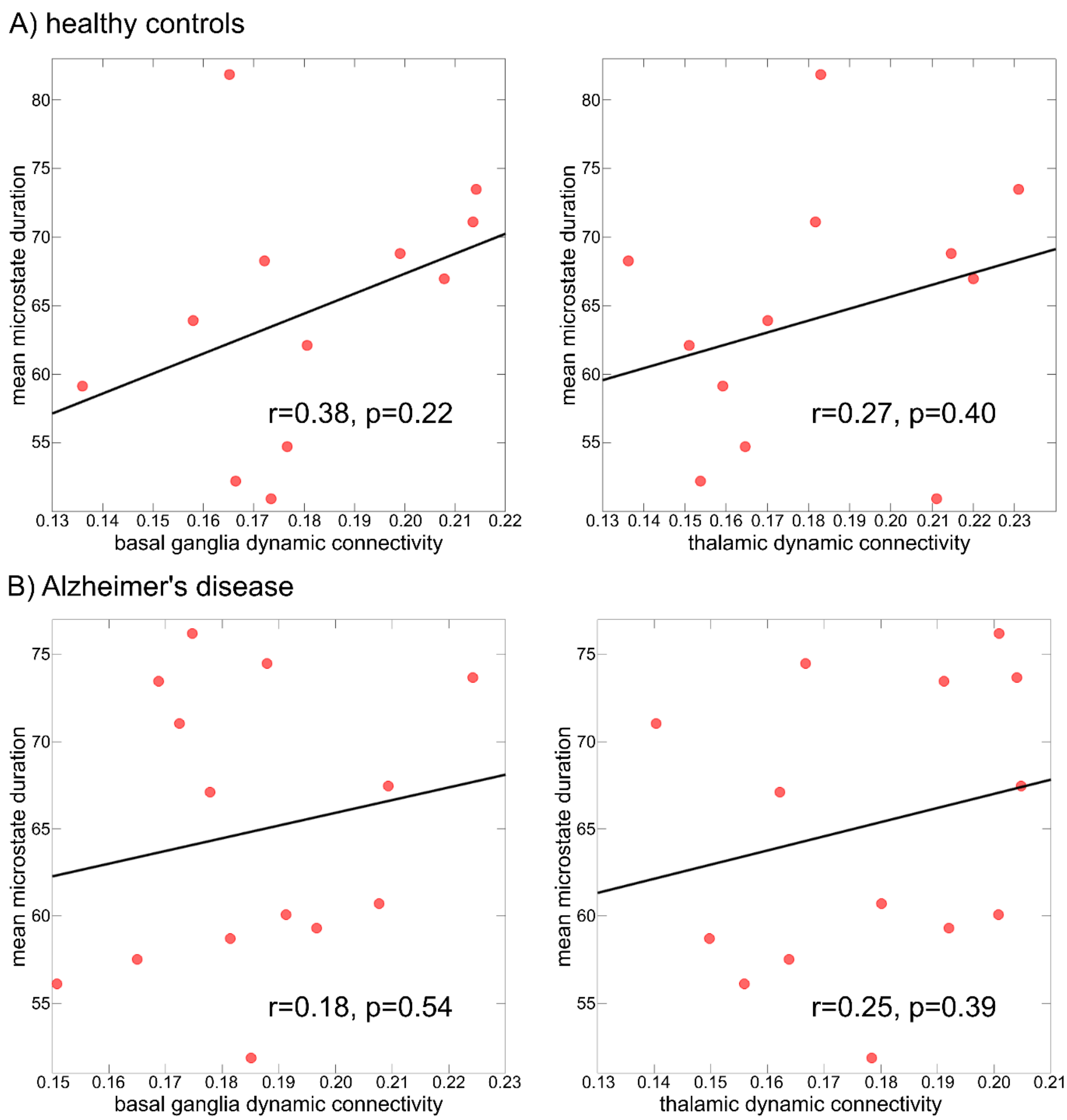

Figure S2: Pearson’s correlation analysis between overall dynamic connectivity of the basal ganglia and thalamic networks in A) healthy controls and B) Alzheimer’s disease.

13. Supplementary references

Abou Elseoud A, Littow H, Remes JJ, Starck T, Nikkinen J, Nissilä J, et al. Group-ICA Model Order Highlights Patterns of Functional Brain Connectivity. Front. Syst. Neurosci. 2011; 5: 37.

Agosta F, Pievani M, Geroldi C, Copetti M, Frisoni GB, Filippi M. Resting state fMRI in Alzheimer's disease: Beyond the default mode network. Neurobiol. Aging 2012; 33: 1564–1578.

Allen EA, Damaraju E, Plis SM, Erhardt EB, Eichele T, Calhoun VD. Tracking Whole-Brain Connectivity Dynamics in the Resting State. Cereb. Cortex 2014; 24: 663–676.

Avants BB, Tustison NJ, Song G, Cook PA, Klein A, Gee JC. A reproducible evaluation of ANTs similarity metric performance in brain image registration. Neuroimage 2011; 54: 2033–2044.

Beckmann CF, DeLuca M, Devlin JT, Smith SM. Investigations into resting-state connectivity using independent component analysis. Philos. Trans. R. Soc. Lond. B. Biol. Sci. 2005; 360: 1001–13.

Biswal BB, Mennes M, Zuo X-N, Gohel S, Kelly C, Smith SM, et al. Toward discovery science of human brain function. Proc. Natl. Acad. Sci. 2010; 107: 4734–4739.

Cerliani L, Mennes M, Thomas RM, Di Martino A, Thioux M, Keysers C. Increased Functional Connectivity Between Subcortical and Cortical Resting-State Networks in Autism Spectrum Disorder. JAMA Psychiatry 2015; 72: 767.

Damaraju E, Allen EA, Belger A, Ford JM, McEwen S, Mathalon DH, et al. Dynamic functional connectivity analysis reveals transient states of dysconnectivity in schizophrenia. NeuroImage Clin. 2014; 5: 298–308.

Kelly RE, Alexopoulos GS, Wang Z, Gunning FM, Murphy CF, Morimoto SS, et al. Visual inspection of independent components: Defining a procedure for artifact removal from fMRI data. J. Neurosci. Methods 2010; 189: 233–245.

Lehmann D, Faber PL, Galderisi S, Herrmann WM, Kinoshita T, Koukkou M, et al. EEG microstate duration and syntax in acute, medication-naïve, first-episode schizophrenia: a multi-center study. Psychiatry Res. Neuroimaging 2005; 138: 141–156.

Poppe AB, Wisner K, Atluri G, Lim KO, Kumar V, MacDonald AW. Toward a neurometric foundation for probabilistic independent component analysis of fMRI data. Cogn. Affect. Behav. Neurosci. 2013; 13: 641–659.

Pruim RHR, Mennes M, van Rooij D, Llera A, Buitelaar JK, Beckmann CF. ICA-AROMA: A robust ICA-based strategy for removing motion artifacts from fMRI data. Neuroimage 2015; 112: 267–277.

Schumacher J, Peraza LR, Firbank MJ, Thomas AJ, Kaiser M, Gallagher P, et al. Functional

connectivity in dementia with Lewy bodies: A within- and between-network analysis. Hum. Brain Mapp. 2018; 39: 1118–1129.

Tomlinson CL, Stowe R, Patel S, Rick C, Gray R, Clarke CE. Systematic review of levodopa dose equivalency reporting in Parkinson’s disease. Mov. Disord. 2010; 25: 2649–2653.
